# Scope and Limitations of Current Antibiotic Therapies against *Helicobacter pylori*: Reviewing Amoxicillin Gastroretentive Formulations

**DOI:** 10.3390/pharmaceutics14071340

**Published:** 2022-06-24

**Authors:** Roberto Grosso, M.-Violante de-Paz

**Affiliations:** Departamento de Química Orgánica y Farmacéutica, Facultad de Farmacia, Universidad de Sevilla, 41012 Sevilla, Spain; rgb1406@hotmail.com

**Keywords:** *Helicobacter pylori*, gastric cancer, peptic ulcer, gastric mucosa, amoxicillin, vonoprazan, gastric inflammation, treatment, antibiotic resistance, gastroretentive drug delivery systems, floating, mucoadhesive

## Abstract

Even though general improvement of quality of life has happened around the globe, statistics show that gastric cancer is still a very serious medical concern in some regions of the world. A big portion of malignant neoplasms that develop inside the stomach are linked to an infection of *Helicobacter pylori*; in fact, this pathogen has already been categorized as a group 1 carcinogen by the World Health Organization (WHO). Still, the efficacy of current anti-*H. pylori* therapeutic approaches is insufficient and follows a worrying decreasing trend, mainly due to an exponential increase in resistance to key antibiotics. This work analyzes the clinical and biological characteristics of this pathogen, especially its link to gastric cancer, and provides a comprehensive review of current formulation trends for *H. pylori* eradication. Research effort has focused both on the discovery of new combinations of chemicals that function as optimized antibiotic regimens, and on the preparation of gastroretentive drug delivery systems (GRDDSs) to improve overall pharmacokinetics. Regarding the last topic, this review aims to summarize the latest trend in amoxicillin-loaded GRDDS, since this is the antibiotic that has shown the least bacterial resistance worldwide. It is expected that the current work could provide some insight into the importance of innovative options to combat this microorganism. Therefore, this review can inspire new research strategies in the development of efficient formulations for the treatment of this infection and the consequent prevention of gastric cancer.

## 1. Introduction

Cancer is one of the major public health concerns in today’s world, especially in developed countries, where it holds second place—just after cardiovascular diseases—in the list of leading causes of death. In 2019, 17.83% of total deaths were attributed to malignant neoplasms, for all ages and both sexes, compared to the data registered for 1990 (12.34%) [1]. For example, in the European Union, this figure increases up to 29% of total deaths in 2019, contrary to the trend found for heart and circulatory diseases. This is one of the main reasons why the biomedical scientific community devotes great effort to finding novel approaches to treat different types of cancer more efficiently, while reducing their associated systemic toxicity. The case of gastric cancer draws attention: there are different regions of the world that can be considered endemic areas for this pathology, mainly due to high exposure to various risk factors, the most relevant being infection by *Helicobacter pylori* (*H. pylori*).

*H. pylori* (initially named *Campylobacter pyloridis*, and later known as *Campylobacter pylori* before its current taxonomical classification [2]) is a Gram-negative bacterium whose very name reveals some other details about its morphology and trophism: the genus *Helicobacter* refers to its shape (of a curved rod), while *pylori* is reminiscent of the pylorus (the opening that connects the stomach and the duodenum, alluding to where the pathogen can settle). It is selectively trophic for the gastric mucosa and its optimal living conditions require lower-than-atmospheric levels of oxygen (microaerophilia) [3]. It is also capable of forming water-insoluble biofilms under certain conditions [4].

Usual therapeutic approaches against this bacterium fall into one of these groups: (a) empiric therapies and (b) susceptibility-based therapies. In any case, the efficacy of current anti-*H. pylori* clinical regimens is deficient and following a worrying decreasing trend that can be confirmed by looking at global data on resistance to key antibiotics. Although amoxicillin appears to be the drug to which *H. pylori* presents the least overall resistance, strains resistant to this and other antibiotics are rapidly becoming predominant, making the development of novel and optimized therapies a matter of utmost urgency. Both the discovery of new regimen-enhancing drugs (such as vonoprazan fumarate) and the generation of smart formulations that improve overall pharmacokinetics have been discussed as potential options to expand the available set of options for *H. pylori* eradication.

This work focuses on reviewing the clinical and biological characteristics of *H. pylori*, especially its link with gastric cancer. Current approaches aimed at improving of the therapeutic repertoire against *H. pylori* will be assessed, and the use of amoxicillin and vonoprazan as a possible dual-cargo option will be considered. In this sense, the works dealing with the development of floating and/or mucoadhesive drug delivery systems for sustained release of amoxicillin cargo will also be reviewed.

## 2. Materials and Methods

In the present review, the Preferred Reporting Items for Systematic Reviews and Meta-Analyses (PRISMA) methodology was used. Searches for scientific papers on the subject in English were carried out in databases such as Google Scholar, Web of Science, Mendeley, Microsoft Academic, Worldwide Science, Science Direct, IEEE Xplore, Springer Link, Scopus, PubMed. The terms used in the research search varied depending on the theme to review (as detailed below) and a general inclusion criterion was applied regarding the research areas of the scientific bibliography: pharmacology pharmacy, infectious diseases, gastroenterology, materials science, chemistry, biochemistry molecular biology, microbiology, science technology, toxicology, polymer science, oncology. In a first step, the abstracts of the publications were analyzed to identify the publications to be reviewed with their full text. In a second step, for the selected articles, a detailed reading of the text and its conclusions was conducted. Other works that were not found in the first search but that were of great interest to the present review and referred to by the selected works were included.

Thus, in the section related to gastric cancer, studies focused on reviewing the status quo of this disease have been included. The terms used for the search were (only applying to title and/or abstract): gastric cancer AND (statistics OR epidemiology OR prevalence) OR “risk factors” OR “*Helicobacter pylori*”. This search was refined by the following inclusion criteria: (a) publication year: from 2022 to 2015, both included, and (b) document type: review. Only the most relevant publications were analyzed. In the section related to characterization of *H. pylori*, publications centered on biological and clinical characteristics were included. The terms used for the search were (only applying to title and/or abstract): “*Helicobacter pylori*” AND characteristics AND infection AND (metabolism OR epidemiology OR physiopathology OR genetics OR “virulence factors” OR “clinical history”). This search was refined by the following inclusion criteria: (a) publication year: from 2022 to 2015, both included. In the section regarding antibiotic-based therapeutic approaches against *H. pylori*, articles related to precedents of therapies used to treat this pathogen were included. The terms used for the search were (only applying to title and/or abstract): (“therapeutic approaches” OR therapy) AND “*Helicobacter pylori*” AND infection AND (empiric OR susceptibility) AND antibiotic. This search was refined by the following inclusion criteria: (a) publication year: from 2022 to 2015, and (b) document type: review. Only the most relevant publications were analyzed. In the section about amelioration of drug combinations, articles focusing on vonoprazan fumarate were included. The terms used for the search were (only applying to title and/or abstract): (“vonoprazan fumarate” OR vonoprazan) AND “*Helicobacter pylori*”. Only the most relevant publications were analyzed. In the sections related to the development of smart, gastroretentive drug delivery systems, studies were included on the use of amoxicillin in *Helicobacter pylori* infections, treatment and formulations, or sustained release of drugs. Formulations in which the antibiotics employed were clarithromycin and metronidazole were excluded. The terms used for the search were: “amoxicillin AND (*Helicobacter* OR *pylori*) AND (“drug release” OR “sustained drug release” OR GRDDS OR formulation) NOT (clarithromycin OR metronidazole)”. This search was refined by the following inclusion criteria: (a) publication year: from 2022 to 2010, both included, (b) document type: articles.

## 3. Gastric Cancer: Stats around the World

It has been estimated that 26,560 new cases of gastric cancer were diagnosed in the United Stated in 2021, more predominantly in males than females, while 11,180 people were predicted to die that same year [5]. This positions stomach neoplasms above laryngeal, ovarian, or esophageal cancer, but with a lower incidence than leukemia, melanoma, or prostate cancer. Moreover, the five-year relative survival rate for gastric cancer (at all stages, in this same country) was estimated to be around 32% based on data collected between 2010 and 2016. This figure is low compared to other types of cancer such as colon (63%), kidney (75%), melanoma (93%), prostate (98%), or breast cancer (90%) [5]. These differences may be related to the fact that the prognosis of this disease is generally poor, being diagnosed in most cases at a late stage, when treatment is not as effective as otherwise desired [6,7].

Some of the risk factors that have been reported for gastric neoplasms are smoking, occupational hazards (rubber industry), exposure to ionizing radiation or asbestos, partial gastrectomy, some genetic polymorphisms, factors related to diet, and infection by *Helicobacter pylori* (*H. pylori*), the latter being the most relevant for the prevention of gastric cancer [8]. Epstein–Barr virus infection and high levels of *N*-nitroso compounds in the body have also been linked to an increased risk of developing this type of neoplasm [9]. Aspirin intake, on the other hand, has been proposed to have a protective role against it [10]. Whether there is a relationship between gastric cancer and peptic ulcers has always been a matter of controversy. Nevertheless, it is known that *H. pylori* infection is not only considered a risk factor for gastric cancer but is also strongly associated with the appearance of other gastric and duodenal ulcer diseases, as reported in 1996 [11]. The authors stated that atrophic gastritis associated with *H. pylori* could evolve into stomach neoplasms or gastric ulcer disease—and, therefore, both pathologies possibly having etiological factors in common—while also establishing that some of the clinical particularities associated with duodenal ulcers could serve as protective factors for gastric cancer [9,12]. In any case, the finding of a stomach ulcer may reveal ongoing colonization of the gastric mucosa by *H. pylori*, indicating an increased risk of the patient’s cells undergoing oncological transformation.

Surprisingly, there are only a few prevention programs for gastric cancer, mainly in East Asia, where this type of neoplasm is highly prevalent. This has obvious historical roots (for instance, differences in dietary factors have been shown to greatly affect the development of gastric cancer) and is therefore linked to what each nation prioritizes medically. For example, the Spanish National Healthcare System provides free screening services for breast cancer (biannual mammography, for women between 50 and 69 years of age since 1990) or for colorectal cancer (biannual fecal occult blood test, for men and women between 50 and 69 years of age, since 2014) [13], which coincides with the fact that the country has high rates for both types of cancer. Conversely, to date, no gastric cancer prevention program has been established in Spain, probably because it does not have such a big impact on society, leading to underdiagnosis of gastric cancer.

**Figure 1:**
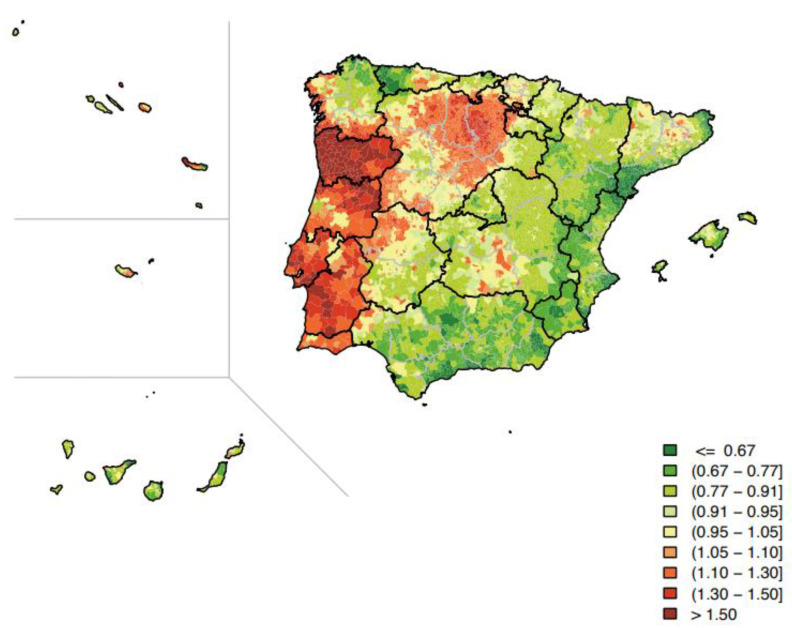
Relative risk of stomach cancer mortality in Portugal and Spain. Adapted from Ref. [8], 2021, Instituto de Salud Carlos III.

In contrast, Portugal, a country that shares all its borders with Spain, behaves differently in terms of stomach cancer, although, a priori, similar rates could be expected for both countries. The recently developed Iberian cancer ‘cartography’ suggests that both countries differ markedly when comparing relative risk of stomach cancer mortality, as if the border served as an impenetrable barrier for risk factors (Figure 1) [8]. In fact, and even though their societies are strongly tied by land, gastric cancer was the 3rd in Portugal, but the 7th in Spain, in terms of mortality in 2018. Therefore, this is an example of how different environmental exposures and ways of living have a crucial bearing on mortality records.

**Figure 2:**
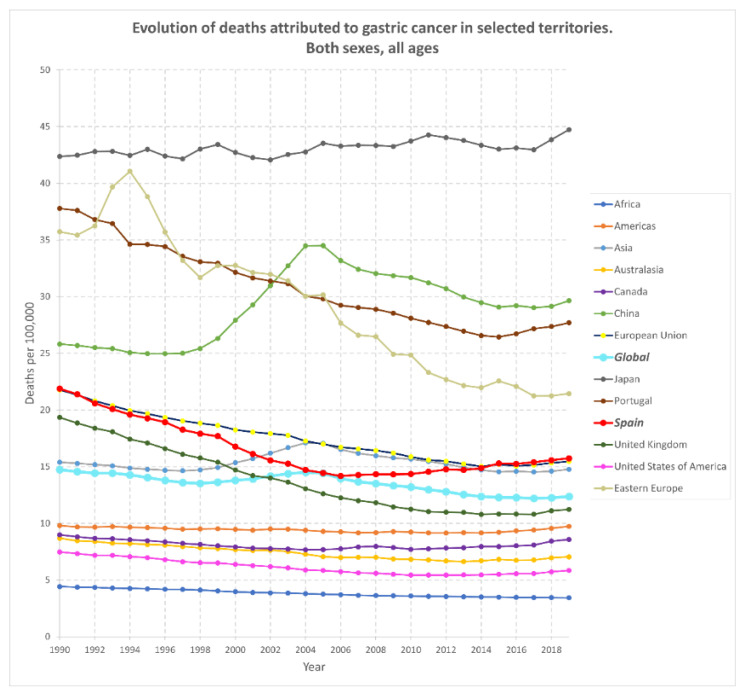
Evolution of deaths attributed to gastric cancer in selected territories (both sexes, all ages). Data obtained from Ref. [1]. 2019, Institute for Health Metrics and Evaluation (IHME), University of Washington.

In any case, the analysis of the evolution of gastric cancer in recent decades (Figure 2) shows that the regions in which the death rates attributed to this disease have exceeded the world average are Asia and Europe (both in the European Union and in Eastern Europe). In fact, these regions may be considered as large endemic areas for gastric cancer. As stated before, poor hygiene standards (especially in developing countries, where *H. pylori* contamination is common), smoking, high alcohol intake, or diets poor in vegetables and greens and rich in smoked and salty food are factors that could justify these data [7,14]. Nonetheless, the global incidence shows a downward trend in the last few years, most likely because of the improvement in hygienic and sanitary conditions worldwide, the progress in food transportation, the promotion of diets rich in vitamin C and fiber, the development of salt-free food preservation methods, and antibiotic treatments against *H. pylori*. Even so, the situation in countries such as Japan is different, where the number of people who died from gastric cancer exceeded the global average by three times in 2019, or Portugal and China, where it almost doubled. The world in general, and these nations in particular, would benefit immensely from any progress in selective therapies against gastric cancer, or more conveniently, from improved diagnostic approaches for its early detection. Furthermore, and considering that *H. pylori* is the main causal agent of this disease, it is also crucial research aiming at detecting its presence in the gastric mucosa as soon as possible and, if that was the case, eliminating it efficiently.

Lastly, gastric cancer can be classified clinically according to several parameters, with the Lauren classification being the most widely used. According to this sorting, there are two main subtypes of gastric cancer—intestinal and diffuse—which differ in genetic characteristics, morphology, epidemiology, or clinical behavior [9]. These differences have an impact on surgical and therapeutic decisions, and therefore, the characterization of each case is an essential need for the practicing gastroenterologist. Moreover, it is important to consider that therapies in development, when in clinical and in vitro trials, should be tested against as many types of cancer cells as possible.

## 4. Clinical and Biological Characterization of *Helicobacter pylori*

The proteome of *Helicobacter pylori* is characterized by the production of catalase, oxidase, and urease [15], the latter being essential for its survival when infecting humans. Urease is an enzyme that catalyzes the hydrolysis of gastric urea into carbon dioxide and ammonia, the latter being responsible for the partial neutralization of gastric acid. By doing so, the pathogen ensures its protection against the harsh environment around it when infecting the stomach (it can only survive when its periplasmic pH is ≥4 [16]), so enhancing its biological success. *H. pylori* also presents several flagella polarly distributed, thanks to which the pathogen can delve into the gastric wall and reach deeper areas underneath the mucus and, thus, avoid direct exposure to the acidic lumen [17]. This all justifies the fact that one of the most impressive characteristics attributed to this bacterium is its ability to persist in the gastric mucosa for years after the first contact, thanks both to these pathogenic mechanisms and to the inability of the patient to eradicate the infection effectively [15]. Indeed, there is evidence supporting that colonization of humans by *H. pylori* has been happening for at least 58,000 years [15], meaning this microorganism has coexisted with our species for thousands of years, probably evolving complementarily.

Clinically, the metabolic transformation of urea by *H. pylori* urease is one of the hallmarks chosen to reliably diagnose this infection, i.e., by the urea breath test. Considering that mammalian cells do not secrete urease, and that bacteria other than *H. pylori* are not usually present in the stomach, exhalation of ^13^C-CO_2_ (or ^14^C-CO_2_) will only be detected when ^13^C-urea (or ^14^C-urea, respectively) is administered to the patients that are colonized by *H. pylori* [18].

**Figure 3:**
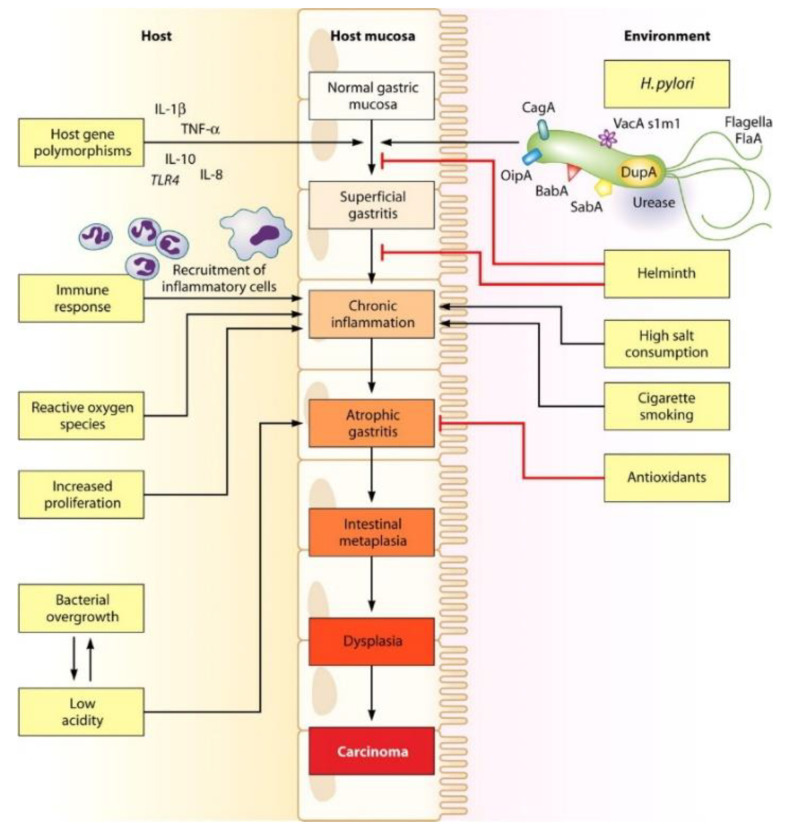
Physio-pathological events associated with an *H. pylori* infection. Reprinted with permission from Ref. [15]. Copyright 2010 American Society for Microbiology Editorial.

This bacterium was first cultured in the early 1980s by Australian researchers Barry Marshall and Robin Warren (eventually awarded the Nobel Prize in Medicine because of this achievement), who isolated it from human gastric biopsies [3]. Previously, the scientific community had reported the existence of spiral bacteria in the stomach epithelium which were capable of surviving those extremely acidic conditions. Nonetheless, it was not until recent years when its colonization of the organ was linked to pathologies related to the gastrointestinal tract (GIT) such as chronic gastritis, peptic ulceration, gastric mucosa-associated lymphoid tissue lymphoma (MALToma) and gastric adenocarcinoma [12,16,19,20]. For several years *H. pylori* was argued to be part of the commensal microbiome of the human species but, considering that all patients infected with it characteristically present an easily recognizable histological pattern of persistent gastric inflammation, it should no longer be regarded as just ordinary flora [12,17]. Main physio-pathological events associated with an infection by this pathogen are represented in Figure 3. Furthermore, it has been reported that this bacterium can wreak havoc in non-GIT regions because it can be involved in the development of iron and/or cobalamin deficiency, or even idiopathic thrombocytopenic purpura [20].

These data are relevant since infection by this bacterium is highly prevalent worldwide (over 50% of the world population are supposed to host it [21]), especially in certain developing regions where sanitary conditions may not be optimal. For example, Africa scores the highest prevalence of *H. pylori* infection among the continents (approximately 70% of the population studied), whereas Australasia has the lowest rates (around 24%) [21]. Interestingly, despite the African figures, Figure 2 reveals that Africa ranks last in terms of gastric cancer cases among the regions studied. This fact may be related to the dietary trends of its population and/or to the fact that it is the region with the lowest life expectancy at birth in the world.

Although many of the infected individuals do not develop any medical symptoms and enjoy a normal life [15], any individual carrying this pathogen is predisposed, even if slightly, to develop *H. pylori*-related gastric imbalances that could range from mildest alterations of the stomach mucosa to life-threatening oncological events [12]. In fact, following these discoveries, *H. pylori* was categorized as a group 1 carcinogen in 1994 by the International Agency for Research on Cancer (part of the World Health Organization, WHO), and it is considered one of the most common etiological agents for infection-related neoplasms [3,15,16]. Several studies have already demonstrated that the risk for gastric adenocarcinoma increases when this bacterium is present in the GIT. It has been estimated that 1–3% of infected patients will eventually develop gastric adenocarcinoma. The latter, in turn, depends on the development of atrophic gastritis after the colonization of the pathogen, its prevalence varying greatly between different areas of the globe [12,15,22]. Indeed, a direct relationship has been established between the presence of the pathogen in the stomach and consequent tumoral transformation in regions such as Europe or Japan, where *H. pylori*-positive gastric cancer cases counted for more than 93% and 99% of the total, respectively [7].

Therefore, eradication of *H. pylori* is today a primary therapeutic objective when detected in the patient’s body, since its elimination significantly changes the natural history of the disease and prevents the escalation of overall risk for stomach neoplasms [12]. Meeting this objective will mean not only a decrease in the incidence of gastric cancer, but also in medical costs, with the consequent benefit for national healthcare systems which will be able to redirect funds towards other critical health needs. In addition, it has been reported that *H. pylori*-related MALToma can be completely cured if pathogen colonization is promptly detected and stopped, thus it is considered as the first clonal lesion that can be eliminated with the prescribing of an antibiotic-based therapy [15].

Regarding the epidemiology of *H. pylori* infection, most colonization begins during childhood, both through interpersonal transmission within families (oral-oral, gastro-oral, or fecal-oral transmission routes), or by intake of foodstuff that was in contact with contaminated sources (food grown in infected soil, *H. pylori*-infected water, etc.), and can persist for decades unless treated [3,12,15,16,19]. Since gastric cancer and *H. pylori* infections are connected, the rapid decline in *H. pylori* transmission rates—by the amelioration of the factors mentioned above—has led to a reduction in cases of gastric cancer and other *H. pylori*-related diseases in the Western world today.

Several genomically diverse strains of *H. pylori* have been classified [15]. To correctly determine the patient’s prognosis, it is essential to identify the main bacterial virulence factors present in the infection. These factors are variable due to the genetic heterogeneity of the microorganism, and most of them are known to have evolved to disrupt host cell signaling pathways. Of note is that the medical outcome of the patient will not only vary depending on these strain-specific factors; the interaction established between the pathogen and the patient’s immune system, as well as possible environmental influences, are also relevantly involved [15,23].

Focusing on bacterial virulence factors, some of them have already been discussed above (production of urease, helical bacterial morphology, flagella-dependent motility). Yet there are others with different functions that can also be highlighted. The expression of proteins such as the blood group antigen-binding adhesin (BabA), the sialic acid-binding adhesin (SabA), or the outer inflammatory protein (OipA) on the pathogen’s outer membrane facilitates its binding to the stomach epithelium and later colonization [15,24]. These proteins potentiate the damaging of the mucosal layer. The *H. pylori* outer membrane protein (HopQ) and the CagY factor are known to act as immune regulators by inhibiting immune activity against the bacterium [23]. On the other hand, the Cag-pathogenicity island (cagPAI) is one of the most relevant virulence elements found in the genome of *H. pylori*. Among other actions, this chromosome region encodes for a multicomponent bacterial type IV secretion system (T4SS) and for an effector protein called CagA, the latter being responsible for the most aggressive behaviors of highly virulent *H. pylori* strains [3,15,19,23,24]. T4SS is necessary for the translocation of CagA into the gastric epithelial cells, where it will widely manifest itself once phosphorylated by cellular kinases. Thus, T4SS acts as a syringe that injects CagA into the stomach epithelium, causing cell elongation and proliferation and promoting expression of proinflammatory IL-8 [23]. The vacuolating cytotoxin (VacA) is also responsible for the pathogenicity associated with *H. pylori* when interacting with gastric epithelial cells. Some authors have reported that VacA has pore-forming properties [25], and is also capable of triggering vacuole formation, cell death (both apoptotic and necrotic), and modification of the autophagic route in the targeted cells [23,24]. Other relevant virulence causes typically found in *H. pylori* are (a) the high-temperature requirement protein (HtrA), which acts as a chaperone and a protease protecting the pathogen from misfolded proteins and collaborating in the intracellular delivery of CagA, and (b) the cholesterol glycosyl-transferase (CGT), which destroys lipid rafts of infected cells and protects bacteria from inflammatory response [19,23].

## 5. Therapeutic Approaches against *Helicobacter pylori*: Precedents

Currently, antibiotic-based therapies against this pathogen are not foolproof whatsoever, especially considering the dramatic increase in antibiotic-resistant *H. pylori* strains. Thus, for example, some studies point out that it is not possible to eradicate the bacteria in at least 2 out of 10 patients treated [12], which may not seem very relevant, but it really is, even more so when resistance to antibiotics is rising exponentially in some parts of the world. This increase in antibiotic resistance is probably due to the use of combined antibiotic therapies that have been routinely prescribed when *H. pylori* was detected in the patient’s stomach.

Recent reviews on the treatment of *H. pylori* classify the therapies against this pathogen into two groups: (a) empiric therapies and (b) susceptibility-based therapies. The former is usually prescribed when the specific clinical characteristics of the strain infecting the patient are not reported, so their objective is to be effective for as broad a spectrum as possible. These typically include either bismuth-containing compounds (as in PeptoBismol^®^, a common commercial brand for bismuth subsalicylate sold in the United States) or rifabutin. Bismuth subsalicylate is frequently used to treat gastrointestinal unwellness (such as traveler’s diarrhea or peptic ulcers [26]) because it effectively displays antiacid and anti-inflammatory properties while being relatively inexpensive. Bismuth salts are also interesting when dealing with digestive problems because their low solubility enables them to exert a mainly local action, thus avoiding systemic absorption [16]. On the other hand, bismuth subsalicylate can cause side effects in patients such as black hairy tongue syndrome or neurological toxicity when its accumulation occurs, mainly in prolonged treatment. Nonetheless, it has been proven to be useful when it comes to stopping colonization by *H. pylori*; it can act as an antimicrobial agent on its own, suppressing the proliferation of the pathogen but not eliminating it [26]. In this way, if combined with the appropriate antibiotics, the overall effect can be the total eradication of colonizing bacteria. Indeed, some studies have reported histologic improvement and overall amelioration of symptoms after prescribing these multiple-drug therapies [26]. Characteristically, empiric bismuth-based therapy is the result of combining a bismuth-containing compound with two antibiotics (tetracycline and metronidazole) plus a proton pump inhibitor (for example, omeprazole or esomeprazole) [12,16,20,27]. Other medical prescriptions may include bismuth citrate instead of bismuth subsalicylate and the results are likely to be similar [12]. In any case, this tactic is regarded as a first-line treatment option for areas where antibiotic resistance is known to be high, especially those with mark resistance to clarithromycin [20]. Still, bismuth-based quadruple therapy is generally ruled out in countries such as Japan, Australia, or Malaysia when treating *H. pylori* because of its side effects [16]. Rifabutin is an antitubercular drug sold under the brand Talicia^®^ [12]. These delayed-release capsules also contain omeprazole magnesium (high dose) and amoxicillin, making them suitable triple therapy against *H. pylori*. This combination is believed to be advantageous compared to others because resistance to rifabutin is rare [3,12]. Nevertheless, overall high-quality scientific evidence for this combination has not been established [27]. Special caution is recommended in the use of rifabutin-containing therapies to avoid both the flourishing of resistant strains of *Mycobacterium tuberculosis* and the risk of myelotoxicity [27].

On the other hand, susceptibility-based therapies generally include three drugs and are indicated if sensibility to specific antibiotics is known after testing the strain infecting the patient. This therapeutic option should not be chosen in any other case to avoid further resistance issues, let alone as empiric therapies, especially considering how high resistance levels already are for the key antibiotics in these combinations [20]. This type of triple prescription usually includes one proton pump inhibitor and two antibiotics, typically clarithromycin plus one of amoxicillin, levofloxacin, or metronidazole [12,16,20,27], based on bacterial culture data. Fluoroquinolones are known to pose a high risk of dire side effects; in fact, the FDA issued multiple warnings between 2008 and 2018 alerting about its use [28]. Therefore, the choice of levofloxacin to treat *H. pylori* is only recommended as a last resort, mainly when the infecting strain is resistant to other antibiotics.

Most of the antibiotics included in anti-*H. pylori* triple therapies require an increase of intragastric pH to function effectively as a bactericidal agent, justifying the addition of a proton pump inhibitor to the combination. Although the addition of the latter can be counterintuitive since the basification of intragastric pH will also make the pathogen proliferate, antibiotics such as amoxicillin need this bacterial growth and take advantage of it. The mechanism of action of amoxicillin and clarithromycin involves targeting a biomolecules that are essential for the bacterial growth phase (either penicillin-binding proteins or 23S rRNA within the 50S subunit of bacterial ribosomes, respectively). This is the reason why these eradication treatments are expected to be successful when the pH inside the stomach rises, since a bigger fraction of the bacteria will be prone to division (thus having greater sensitivity to these antibiotics) instead of remaining inactive (thus resisting antibiotics) [29,30]. It has also been reported that proton pump inhibitors have antimicrobial activity against *H. pylori* per se [16], and may even hinder its urease activity [30], causing the prescription of these compounds to evolve from simple administration for symptomatic relief to actually help enhance the overall pharmacologic attack. All in all, medical data seem to support the idea that the synergy between proton pump inhibitors and antibiotics is necessary (or at least beneficial) for the elimination of *H. pylori*.

Another important aspect to bear in mind is that most of the anti-*H. pylori* chemicals described above (metronidazole, levofloxacin, and clarithromycin) can be classified as concentration-dependent drugs [16], i.e., the extent of microorganism elimination is a function of the antimicrobial concentration (they will have a more powerful effect the higher their concentration gets). In contrast, amoxicillin stands out as a time-dependent drug in this pathology [31], thus being more effective the longer its concentration surpasses the established minimal inhibitory concentration (MIC) for *H. pylori*. Therefore, regarding amoxicillin, instead of aiming to achieve higher drug levels of the drug, attention should be focused on prolonging the time interval in which the antibiotic concentration remains above MIC, since this is the only PK-PD parameter that correlates with the efficacy of beta-lactam antibiotics [32]. Taking advantage of this unique characteristic of amoxicillin may be crucial to improve current therapeutical approaches when treating *H. pylori* colonization.

On another front, for both empiric and susceptibility-based therapies, optimal anti-*H. pylori* treatment lasts 7 to 14 days. These treatments are typically longer in the United States (10–14 days) than in Europe or Asia (weeklong approaches) [12,16]. Although susceptibility-based approaches are preferred due to their more targeted antibiotic usage, advances in these therapies must be accompanied by improvements in methods for assessing antibiotic resistance of infecting strains. Today, only a few techniques, including cultures or polymerase chain reactions, are available to determine it. Furthermore, susceptibility results do not necessarily translate to complete elimination of the bacteria in vivo. Indeed, a 2020 meta-analysis concluded that the evidence is insufficient to discourage empirical regimens in routine clinical practice and make susceptibility-guided treatment the mainstream of future therapy, at least until susceptibility testing is optimized and globally accessible [33]. Results of opposite cost-effectiveness (in favor of the empirical method) and similar efficacy between the two therapeutic approaches have also been found. Consequently, the development of inexpensive and readily available tests to determine antibiotic resistance appears to be essential for susceptibility-based therapies to become first-line treatment in *H. pylori* infection.

Other pharmacological treatments, such as concomitant, hybrid, and sequential therapies [12], have been attempted in recent years but are no longer recommended due to mounting evidence showing little success [27].

Sequential therapy against *H. pylori* gets its name from the fact that it is based on a two-stage medical regimen: first, one proton pump inhibitor and one antibiotic (usually amoxicillin) are prescribed for 5 to 7 days; and second, amoxicillin is replaced by two other antibiotics (such as clarithromycin and metronidazole) and medication is continued for an additional 5 to 7 days [16,20]. Sometimes a fluoroquinolone (levofloxacin) is also included the second course of medication [34]. In essence, it can be seen as quadruple therapy, or even triple therapy if amoxicillin is repeated in the second period. Evidence regarding greater success of this therapeutical approach compared to others is limited, contradictory, and often regarded as low-quality [27,34]. Indeed, as of 2017, the FDA had not approved this therapeutical regimen [34]. Overall, sequential therapy has been relegated to a conditional first-line choice, but only in certain geographic areas and under specific circumstances (i.e., to treat patients infected with single clarithromycin-resistant strain [20]), and without substantial evidence in any case [27,34].

Concomitant *H. pylori* therapy (also known as non-bismuth quadruple therapy) is prescribed for 10 days and contains three antibiotics (amoxicillin, clarithromycin, and metronidazole), with the hope that the infecting strain is sensible to, at least, one of them. One bismuth-free proton pump inhibitor is also prescribed. The number of pills to be taken by the patients is greater than normal, which makes optimal adherence to this approach more cumbersome than in the other cases [20]. Out of the all the discouraged options, concomitant therapy is the one that can still be seen in use today, even being recommended as an alternative first-treatment choice in areas where the prescription of bismuth derivatives is not allowed, or clarithromycin resistance is prevalent. Some studies have even reported concomitant therapy to be equal or more effective than standard triple therapies or sequential approaches when dealing with *H. pylori* [16]. Nonetheless, in the case of concomitant therapy, treatment costs are higher and a disproportionate exposure of patients to multiple antibiotics is promoted. A 2020 meta-analysis reported that the efficacy of bismuth-containing quadruple treatment surpasses that of concomitant therapy [35], therefore making the former an equally efficient alternative to the latter with fewer antibiotics.

Hybrid therapy against *H. pylori* consists of two dosing periods of 7 days each; the first includes one proton pump inhibitor and amoxicillin, while during the second one two more antibiotics (clarithromycin and a nitroimidazole) are added. Several studies have been conducted comparing the efficacy of hybrid therapy to sequential or concomitant therapies, and none of them have reported significant differences [20]. A 2015 meta-analysis noted that this therapeutical regimen appeared to be more successful and equally tolerable than clarithromycin-based 7-day triple therapy [36], leading the American College of Gastroenterology to suggest it as an alternative treatment in some cases. Hybrid therapy efficacy in areas with high clarithromycin and metronidazole resistance (such as some Mediterranean countries) has also been reported [27].

In summary, medical prescriptions have included up to four drugs—of which, usually, two or three are antibiotics—with the aim of eliminating the pathogen without knowing on occasion if it was susceptible to them. The relative success of these packaged therapeutic regimens cannot compensate for the fact that some of the drugs present in these combinations are probably unnecessary, this being a decisive factor for the failure of *H. pylori* eradication today. Table 1 shows the data on resistance to the main antibiotics used in anti-*H. pylori* therapies, retrieved from studies carried out in different regions of the world. The information was selected considering the latest and most complete data available for each area. Even though this type of report varies greatly depending on the studied parameters, the increased prevalence of resistance to key antibiotics used in anti-*H. pylori* therapy is evident, thus limiting the applicability of traditional regimens [16,37]. This is not a problem unique to *H. pylori*: a review published in 2020 revealed that more than 80% of the articles studied reported over 60% dispensing of antibiotic without a prescription [38], which means that the latter is far more common than over-the-counter drug dispensing in other medical fields. This is causing antibiotic resistance to emerge as one of the major health problems worldwide. Optimization of current therapeutic approaches is becoming urgent as the massive flourishing of resistant strains must be stopped. Thus, in rethinking this issue, the medical community must be concerned with (a) reducing the number of antibiotics prescriptions when possible, and (b) adapting antibiotic-based treatments to local resistance patterns.

Overall, the outcome obtained after the analysis of the degree of efficacy of anti-*H. pylori* treatment regarding traditional approaches (especially the triple regimen) are no longer acceptable under the Maastricht consensus, which an anti-*H. pylori* therapy acknowledged as useful if it is able to achieve an eradication rate of 80% or more [39].

**Table 1:** Percentage of *H. pylori* strains studied showing primary resistance to key antibiotics in anti-*H. pylori* therapies around the world.

	*Region*	Africa [40]	Australasia [41]		Americas [42]	Northern Europe (Norway) ^b^ [43]	Southern Europe ^b^ [43]	Asia					World [44]
								Vietnam [45]	Russia [46]	Iran [47]	China [48]	Continent [49]	
*Antibiotic*		1986–2017	pre-2000	2000 Onwards	2007–2017	2013–2020		2000–2016	2011–2020	2010–2020	2013–2020	2006–2009	2015–2017
Clarithromycin		29.2	6.46	16.1	10	7.0	28.0	34.1	10.4	25.3	55.2	18.9	27.2
Metronidazole		75.8	50.1	50.5	23	26.0	30.5	69.4	34.0	64.9	68.0	37.1	39.7
Levofloxacin		17.4 ^a^	N/A	2.92 ^a^	15	2.5	23.5	27.9	20.0	21.9	49.7	11.6	22.5
Amoxicillin		72.6	0.13	2.09	10	0	0.20	15.0	1.35	20.7	0.7	11.6	4.55

^a^: Refers to fluoroquinolones in general; ^b^: Refers specifically to naïve patients with *H. pylori* infection.

Focusing on the European continent (Table 1), it is surprising to see how disparate the rates of resistance to clarithromycin are between the north and the south of the continent. Some authors correlate this to the different macrolide prescription between countries. The case of Spain is significant, where it was reported that up to 49% of *H. pylori* strains had lost sensibility to clarithromycin, probably because macrolides were used very loosely in the treatment of infections during the 1990s. In contrast, northern European nations appear to be subject to more stringent regulations, explaining, for example, the emergence of only 1% resistance to clarithromycin in the Netherlands [20]. Some western European countries have also reported to having up to 45% of *H. pylori* strains resistant to metronidazole, but none of these reach the level seen in the African continent or in some areas of Asia. These differences can be attributed to frequent prescription of metronidazole as an antiparasitic and as a cure for gynecological infections [20]. Analogously, high levofloxacin resistance levels may be related to the recurrent use of fluoroquinolones to treat urinary tract infections [20]. In conclusion, of all the antibiotics analyzed previously, amoxicillin (AMOX) appears to have the lowest prevalence of resistance among *H. pylori* strains (except in Africa), therefore making it an ideal candidate in optimized dual therapy (amoxicillin and a proton pump inhibitor) to be studied as a first-line treatment for infections in most countries.

Current anti-*H. pylori* medical approaches face other problems, one of them being patient adherence to therapy. Since most therapies against *H. pylori* have so far required the combination of several drugs, the discomfort experienced by the patient due to multiple-pill treatments often reduces compliance. Few studies have been conducted on ways to increase the latter and, thus, optimize clinical outcomes when treating infection by *H. pylori* [50]. One means is to optimize therapeutic regimens so that the number of pills is minimized while efficacy is maintained. Another possibility is the prescription of formulations such as Talicia^®^, one capsule that contains all the active pharmaceutical ingredients (API) necessary for the treatment. On the other hand, not only a simpler therapeutic regimen but also the use of drugs with fewer adverse effect would lead patients to fully adhere to the treatment plans [50]. The use of probiotics as beneficial adjuvants in antibiotic treatment has been proposed, since they may reduce certain associated side effects, especially diarrhea. In addition, professional counseling, ongoing follow-up, and comprehensive explanations of the risk-benefit ratio of medication observance are needed to make the patients aware of the importance of adhering to their medication regimen, even when side effects may be uncomfortable [51].

Patient’s ethnic background also plays an important role in the efficacy of the treatment since some polymorphisms are known to impact negatively on therapy success. For example, genetic variations in cytochrome P450 isoenzyme 2C19 (CYP2C19) allow classification of patients into three main groups: extensive metabolizers, intermediate metabolizers, and poor metabolizers. This is relevant because CYP2C19 is the responsible for metabolizing (thereafter, eliminating) proton pump inhibitors, thus altering their pharmacokinetics and drug residence time, and, consequently, determining how high the intragastric pH gets [37,52,53]. Therefore, significant differences in gastric acidity between the metabolic groups have been reported. For example, when administering 40 mg of rabeprazole, the pH ranged from 5.9 (among poor metabolizers) to 4.3 (among extensive ones) [16]. Loss of function in this enzyme-encoding gene is relatively common, for example, in Thailand, which makes its population predominantly poor metabolizers for drugs such as omeprazole or lansoprazole, thus achieving a more effective inhibition of the secretion of stomach acid, and eventually stopping *H. pylori* infections more efficiently [34]. In other populations, this issue can be easily solved by using proton pump inhibitors whose efficacy is less likely to be affected by CYP2C19 (such as esomeprazole) or not affected at all (such is the case of the novel drug vonoprazan). Another answer that has been proposed is the prescription of lower but more frequent doses of proton pump inhibitors, even if they are clear targets of CYP2C19; by doing this, intragastric pH was shown to be maintained at significantly less acidic values, regardless of CYP2C19 variations [16].

Variations in interleukin-1β and interleukin-1RN genotypes have also been linked to differences in drug response against H. *pylori* [52]. These cytokines can act as endogenous inhibitors of gastric acidic secretion, allegedly increasing the likelihood of effective eradication of the bacteria when administering any proton pump inhibitor [16]. In this way, patients with the interleukin-1β-511 T/T genotype have shown higher eradication rates than those with interleukin-1β-511 C/T or C/C genotypes, and the same has been reported for patients with the interleukin-1RN 1/2 genotype in contrast with those with interleukin-1RN 1/1 genotype [16,52,53]. All in all, amelioration of therapies against *H. pylori* is required to overcome these drawbacks.

## 6. Solution I: Amelioration of APIs Combinations

Numerous approaches have been proposed to eliminate *H. pylori* with probiotic supplementation and phytomedical regimens [54]. Nonetheless, their potential remains to be demonstrated, especially considering that most of the data were obtained from animals or in vitro, but not from humans [16]. Probiotics are administrated in limited amounts to take advantage of their beneficial properties on the gastrointestinal niche, i.e., their anti-inflammatory and anti-oxidative effects [20]. Some research groups have studied the combination of probiotics, such as *Saccharomyces boulardii* or different *Lactobacillus* sp. strains, with antibiotics. Even though antibiotic side effects, such as diarrhea, decreased significantly, the eradication rate did not change substantially [16,20,27,34,37,54].

More interestingly, vonoprazan-based therapies have also recently been explored. Vonoprazan fumarate (VONO, also known as TAK-438; hereinafter referred to as “vonoprazan”) is a novel drug that can act as a potassium-competitive acid blocker (P-CAB), that is, a chemical capable of competing with potassium, and reversibly bind to the ATPases found in parietal cells that are responsible for secreting acid to the stomach [29,55,56]. It differs from other P-CAB in its chemical structure, with the absence of some moieties found in other P-CABs that are known to be hepatotoxic and blockers of cardiac potassium channels [56,57,58,59]. Pharmacologically, this drug provides a more sustained, robust, and dose-dependent inhibition of gastric acid secretion compared to ordinary proton pump inhibitors. Its accumulation within the canaliculi of the target cells is achieved more fruitfully than that obtained by regular proton pump inhibitors thanks to its enhanced stability in acidic media and its relatively alkaline character (pKa ≈ 9) [3,29,56,58,59].

VONO choice is also advantageous from a pharmacokinetic point of view. Firstly, and contrary to ordinary proton pump inhibitors, it does not act as a prodrug, which explains its quicker, longer-lasting, and more effective suppression of acidic secretion [29,57,58,59]. Indeed, maximum pharmacological effect is reached from the first dose, while proton pump inhibitors require at least 3 days of consistent administration to achieve a steady-state concentration and optimal clinical effects [56]. Secondly, metabolic elimination of vonoprazan has been reported to be mainly linked to CYP3A4/5 enzyme, instead of CYP2C19 [56,57,59,60], the encoding genes of the first ones being more stable among people from different geographical areas. Thus, elimination of vonoprazan is not affected by polymorphisms related to metabolic enzymes (as was the case with regular proton pump inhibitors) and, hence, overall therapeutic outcome is not dependent on interindividual genetic variability [59]. Thirdly, it can be ingested with food or under fasting conditions unlike traditional proton pump inhibitors, which are recommended to be administrated without food to increase their bioavailability [56,57,59]. Fourthly, VONO plasma half-life is considerably longer (about 7 h for a 20-mg dose) than that of key proton pump inhibitors (less than 2 h), allowing for a clinical regimen more spaced in time and, hence, more convenient [57,59]. Lastly, the administration of VONO within a protective coating in not necessary (unlike omeprazole, for example) since its pharmacological activity is not reduced when exposed to gastric acidity [54], nor are its pharmacokinetics significantly affected by gastric peristalsis [56]. Notably, VONO has been reported to perform efficiently: when monitoring intragastric pH after its administration, pH values remained ≥ 4 for over 24 h post-dose [55,57,60]. The main downside of VONO, when compared to proton pump inhibitors, is that it does not exert antimicrobial activity against *H. pylori* per se [29]. Moreover, elevated incidence of gastric endocrine tumors in rodents has also been reported when exposed to massive doses of VONO [59]. Nevertheless, this should not be a concern for clinical use, especially when considering the propensity of these animals to develop such neoplasms when subjected to acid suppression treatments. It is also likely that the administration of VONO promotes the overgrowth of the intestinal microbiota due to the very nature of its pharmacological activity (suppression of acid secretion), as occurs with proton pump inhibitors [59].

Consequently, VONO is one of the most promising inhibitors of acidic secretion to date. Substitution of conventional proton pump inhibitors by VONO is likely to overcome the limitations of such drugs and improve the effectiveness of anti-*H. pylori* treatments, while reducing the number of antibiotics in the regimen. This drug was already marketed in Japan in 2015 under the trade name TAKECAB^®^ (co-promoted by Takeda Pharmaceutical Company Ltd. Tokyo, Japan, and Otsuka Pharmaceutical Company Ltd. Tokyo, Japan) [61] and is currently also sold in several Asian and South American countries [57]. Relevantly, in a 2021 clinical trial for *H. pylori* eradication, VONO-based triple therapy was compared with omeprazole-containing triple therapy. Both success rates and side effects were reported to be similar, but the first option was optimal with a treatment duration of 7 days versus the 14 days required in the other case [62]. Additionally, dual therapy combining low-dose AMOX and VONO has been tested with adequate results [63]. This combination has proven to be not only as safe and tolerable as traditional triple therapy, but also an even more successful treatment in terms of *H. pylori* eradication and reduction of antibiotics misuse [29,55,63]. However, larger-scale studies evaluating it in other populations are needed.

Regarding Europe and the United States, clinical trials are being conducted where VONO is the protagonist [59]; most of them focus on monitoring the safety and pharmacokinetic parameters of VONO, or its use as a better therapeutic alternative for gastroesophageal reflux disease. At present, only a few works have investigated its incorporation in treatments for *H. pylori*-infected patients (and mostly with populations from the UK or France) [55,57,58,64]. There has been a delay in conducting clinical trials with VONO in Europe and North America, compared to Asian countries such as Japan. The reason may be due to the lower prevalence of gastric cancer caused by *H. pylori* in both regions compared to Asia, where this is a pressing medical concern. While there is a need to evaluate vonoprazan-based regimens where populations are mainly made up of members of other ethnic groups and where antimicrobial resistance follows different patterns, the results are not expected to vary significantly from those obtained in Asian countries, according to a study conducted in 2022 [65]. In said investigation, a population pharmacokinetic analysis was performed to assess the impact of selected variables on exposure to vonoprazan. The results suggested that variations in weight, age, or race would not have a clinically significant impact on the safety of vonoprazan, and the efficacy and safety data for vonoprazan in Asian countries are expected to be translatable to non-Asian populations [65]. Additionally, the results of a phase III clinical trial that incorporates vonoprazan in commercial formulations in the USA and Europe have recently been published. This study concluded that triple and dual vonoprazan therapies (vonoprazan plus amoxicillin plus clarithromycin, or vonoprazan plus amoxicillin, respectively) were superior to lansoprazole triple therapy (lansoprazole plus amoxicillin plus clarithromycin) in all patients evaluated, especially in those infected with a clarithromycin-resistant *H. pylori* strain. Furthermore, the side effects associated with any of the pharmacological treatments containing vonoprazan were not greater than those found with classical therapy. These results suggest that commercialization of vonoprazan in Europe and the USA is imminent [66].

To conclude, in the search of optimal treatments of *H. pylori*, the choice of amoxicillin as the antibiotic accompanying vonoprazan may be logical, since it is the one with the least global resistance according to recent data (Table 1). Conversely, clarithromycin has been shown to be a potent inhibitor of CYP3A4, so its combination with vonoprazan can cause serious metabolic disorders [67]. Consequently, if the AMOX-VONO dual approach proves to be successful, it could be a major step forward in the treatment of *H. pylori* infections since only a low dose of a single antibiotic would be needed.

## 7. Solution II: Development of Smart, Gastroretentive DDSs

Other approaches have focused not on the therapeutic compounds prescribed, but on creating drug delivery systems (DDSs) suitable for the optimal administration of target APIs. This is the case of the lipid nanocarriers designed for the encapsulation of clarithromycin, which later were incorporated into microcapsules, in a study published in February 2022 [68]. Effective antibiotic release from this gastroretentive and pH-sensitive DDS was achieved, thus offering an alternative to optimize anti-*H. pylori* treatments. Another example is the amoxicillin-loaded, pectin-coated liposomes developed by Gottesmann and coworkers [69]. Pectin, polysaccharide obtained from apples, has been reported to interact with both porcine gastric mucins (thus making this formulation gastroretentive) and some of the adhesive proteins expressed in the surface of *H. pylori* (such as BabA, inhibiting them). The system prepared (a) could dock to the stomach mucus, (b) avoided *H. pylori* colonization by hindering its adhesion to the organ, and (c) killed the remaining bacteria thanks to release of AMOX. These works demonstrate that investigation in gastroretentive drug delivery systems (GRDDSs) is a promising method that can render fine-tuned formulations with appropriate features as will be describe below, even more if considering that the same idea can be expanded to other relevant APIs and other DDSs.

Among the all drugs used, AMOX is probably the most promising one to use in these systems since low amoxicillin-resistant *H. pylori* strains have been reported worldwide, as stated before. Optimal administration of AMOX would require small and frequent doses considering its time-dependent pharmacodynamic [16], so its prescription inside a sustained-release DDS could facilitate *H. pylori* eradication, accelerate clinical amelioration, and promote patient’s adherence to the regimen. Such DDSs include gastroretentive systems, which will be detailed next.

### 7.1. Gastroretentive Formulations for the Sustained Release of Amoxicillin in the Stomach in Helicobacter pylori Treatment

Oral administration is the most widespread route for drug intake, accounting for about 90% of all therapies prescribed [70]. Oral formulations have several relevant advantages such as (a) allowing easy storage, transport, and administration, (b) being non-invasive, and (c) promoting high patient compliance. Nonetheless, they also display crucial drawbacks, especially when the fluctuating nature of the GIT is considered. Physiological factors (i.e., pH, gastric emptying time, interactions with food, etc.) are known to affect the bioavailability of oral drugs and significantly alter the dose effectiveness.

As mentioned above, *H. pylori* resides in the gastric mucus layer at the mucosal-epithelial cell interface. The antimicrobial drug access to this site of infection is restricted both from the stomach and the gastric blood supply [71]. Choosing API formulations to exert their action locally has been shown to be better at eradicating this pathogen from the stomach. That is why the scientific effort is fundamentally directed towards the development of formulations that localize the antibiotic of choice in the gastric region to yield greater bioavailability, thus improving therapeutic efficacy [72].

However, in the treatment of infections caused by *H. pylori*, the failure of a single antibiotic therapy could be due to poor stability of the drug in the acidic pH of the stomach, poor permeability of antibiotics across the mucus layer, or even the non-availability of therapeutic antibiotic concentrations at the infection site soon after oral administration of a conventional dosage form [73]. All this adds up to other factors previously discussed in this work, such as the exponential increase in resistant *H. pylori* strains. Consequently, prolonged local release of drug is needed to diffuse sufficiently to the bacteria and to optimize antibiotic use in this kind of therapy.

Gastroretentive drug delivery systems (GRDDSs) have emerged as an ideal approach to overcome these challenges, for example, in local treatment of gastric *H. pylori* infections. They are designed to lengthen the gastric residence time (GRT) and drug release into the bacterial surroundings, thus reducing the frequency of medication and the doses required to cure the patient [74]. Other aspects where GRDDSs can play a key role could be the prolongation of the release of drugs with narrow absorption windows, or the sustained liberation of APIs soluble at acidic media [75].

Among these GRDDSs, several methodologies have been attempted to improve the sustained and local release of AMOX in the treatment of *H. pylori* infections, low-density (floating) systems (which include the raft-forming formulations) [76] and mucoadhesive devices [75] being the most promising approaches.

### 7.2. Floating Formulations

The design of buoyant systems is one of the main approaches to achieve effective GRDDSs [76], thus having a bulk density lower than that of gastric fluid (1.004 to 1.010 g/mL). The types of systems that have been designed as AMOX-GRDDSs go from floating in situ gelling system [77,78] to floating beads [79], bilayer floating tablets [80], hollow tablets [81], floating capsular devices obtained by 3D printing [82], or floating raft systems (FRSs) [83], and floating microballoons/spheres [84] (Table 2).

In general terms, the overall floating behavior of the dosage form can be reached by addition of swelling enhancers or wicking agents [85], as well as the incorporation of effervescent combinations [86]. Among the methods employed to make AMOX-loaded systems float, the one most frequently used is the inclusion of gas generating combinations (effervescent mixtures), so the formed gas gets entrapped into the formulation. This would lead to increasing the free volume in the system and hence, reduce the overall density of the GRDDS and make it float. These gas forming mixtures usually include a sodium or calcium carbonic acid salt [76], which can be dissolved in the acidic medium of the stomach and dissociated into their ions. Gastric acidic environment can promote the protonation of these anions (carbonate or bicarbonate anions) to render carbonic acid, that will subsequently decompose into CO_2_ (gas) and water. Due to the administration of proton pump inhibitors in *H. pylori* therapies, the pH of the stomach may not be acidic enough to generate the abovementioned reaction and therefore, a proton donor molecule (such as citric acid) is generally included in the formulation.

The most common CO_2_-generating salts employed in such formulations are sodium bicarbonate (NaHCO_3_) in combination with sodium citrate [78] or citric acid [80,87]. The use of the low-soluble-at-neutral-pH salt CaCO_3_ is restricted to those systems in which a carboxylate polymer is used. In those cases, not only do the carbonate ions generate CO_2_, but also the Ca^2+^ cations act as an ionic cross-linker between the carboxylate groups, improving the final stability of the formulations. For example, CaCO_3_ was included in the formulation for the formation of AMOX-loaded floating and mucoadhesive sodium alginate-based (NaAlg) microspheres [88], whereas CaCO_3_ and citric acid were used for in situ-gelling systems based on gellan gum (G) [77]. This methodology has been followed for the design of new approaches in the treatment of *H. pylori* in which an anionic polymer and CaCO_3_ are involved; for example, in the preparation of clarithromycin-loaded (CLA) gellan gum-based floating beads [79] or in FRSs loaded with metronidazole (Mz), in which the formulation includes both anionic polymers: G and NaAlg [89]. Furthermore, the formation of gas can stem from the slow evaporation of a volatile solvent enclosed in the formulation; such it is the case of the slow evaporation of dichloromethane in AMOX-loaded microballoons in the drying procedure during their manufacture [84] (Table 2).

Another alternative to impart floatability to AMOX-based formulations is, on the one hand, the use of low-density lipids such as sunflower oil [72] or light mineral oil in oil-entrapped buoyant beads [90]. Moreover, lipids such as glyceryl monostearate (GMS), Precirol^®^ (PRE) and Compritol^®^ (COM) have also been used as floating assistant agents in Mz formulations [89]. On the other hand, the design of hollow devices is aimed for: for example, hollow tablets for the administration of AMOX and CLA [81], or 3D-printed capsules for the inclusion of a conventional AMOX capsule into it [82].

Regarding floating lag time (FLT), this parameter is close to zero when a hollow device is used, such as either capsular [82] or modular tablets [81]. It was also observed that the use of gas-generating mixtures based on NaHCO_3_ with citric acid derivatives [78,80] displayed very short FLTs (≤40 s) compared to systems in which CaCO_3_ [79,83] is chosen. This could be related to the slow solubility of the latter, which is enhanced at acidic pH.

Interestingly, and to the authors’ knowledge, all the formulations proposed for the manufacture of floating AMOX-loaded GRDDSs incorporate at least two or three additional excipients to guarantee such buoyancy during the expected release tempo, and there is no example in which its floatability is determined by its own microstructure. This excipient-containing approach is costly and has an ecological impact that should be prevented in future GRDDS designs.

Regarding AMOX release studies from floating devices, it is notable that the formulations that render the optimal FLT (hollow modular tablets or hollow imprinted devices) are at the same time the ones that exhibit the less effective drug retention, with up to 90% of cumulative drug release within 1.5 h [81,82]. Conversely, adequate control on AMOX release was found in some cases, with cumulative drug release ranging from 34% to 97% in 8 h [84]. Burst release (>40% in 1 h) is quite common in floating gel systems [78] and, although an initial peak release could be of help to provide the MIC, this parameter must be controlled for the final formulation (Table 2).

**Table 2:** Selected AMOX-loaded floating formulations used in the treatment of infections by *H. pylori*.

Drug	Formulation	Matrix-Forming Polymers	Other Components	Floating Time (FT) Floating Lag Time (FLT)	DL (%) EE (%)	Sustained Release: Time (h), and Cumulative Drug Release (%)	Preparation Method	Ref.
AMOX	Floating oral in situ gelling system	NaAlg HPMC K100 (thickening agent)	CaCl_2_ (Xrlinker), sodium citrate, NaHCO_3_	>24 h FLT ≤ 30 s	7.5% (*w*/*v*) …	pH 1.2: Burst release of drug >40% in 1 h 66–85% in 6 h	In situ gelation by Ca^2+^ ions	[78]
AMOX	Bilayer floating tablets	*Aloe vera* gel powder HPMC K4M, HPMC K100M	NaHCO_3_, Citric acid	>8 h FLT: 24–36 s		pH 1.2: 97% in 8 h	Prepared by applying direct compression technique	[80]
AMOX + CLA	Floating modular DDS (hollow tablet)	- For AMOX: HPMC K100M - For CLA: HPMC K15M, PVP K30, PEG 6000	Talc, magnesium stearate	5 h FLT: 0 s	AMOX: 65% CLA: 84% …	- AMOX at pH 1.2: ~55% in 3 h - CLA at pH 3.0: 75–90% in 3 h	* Direct compression for AMOX * Compression of CLA-loaded granules	[81]
AMOX	Floating 3D-printed capsular devices	PVA filaments	BaSO_4_	* In vitro: FT = 14 h; FLT = 0 s * In vivo (rabbits): FT = 10 h	--	pH 1.2 *ca.* 80–100% in 1.5–3 h	Fused deposition modeling (FDM) 3D print 3D printing and thermal crosslinking HME-3D	[82]
Mz	FRS	(1) NaAlg (2) G	COM, PRE, GMS Sodium citrate and CaCO_3_	>24 h TLF: 1 min		pH 2: 75–90% in 4–6 h	Raft systems prepared by ionotropic gelation	[89]
AMOX	FRS	GG	GMS (lipid phase) CaCO_3_ + Sodium citrate (Xrlinker, gas generating agent)	FLT: 1–5.5 min	…	pH 1.2: 80–97% in 24 h	Emulsion and ionic crosslinking method	[83]
Luteolin	Floating microsponge	Eudragit SR100, EC	Tween 80 emulsifier	>8 h FLT: 0 s	--	pH 1.2: 20–50% in 12 h	Quasi-emulsion method	[91]
AMOX	Floating microballoons	CAP Eudragit S100	PVA Mixture of CH_2_Cl_2_, EtOH, iPrOH	Buoyancy: 43–96%	… EE = 57–93%	pH 1.2 34–75% in 8 h	Emulsion-solvent diffusion method	[84]

AMOX: Amoxicillin; CAP: Cellulose acetate phtalate; CLA: Clarithromycin; COM: Compritol^®^; Cr: Compritol ATO 888; DL: Drug loading; EC: Ethyl cellulose; EE: Encapsulation efficiency; FLT: Floating lag time; FT: Floating time; FRS: Floating raft systems; G: Gellan gum; GG: Guar gum; GMS: Glyceryl monostearate; HPMC: Hydroxypropylmethyl cellulose; Mz: Metronidazole; NaAlg: Sodium alginate; PRE: Precirol^®^; PVA: Polyvinyl alcohol; XrL: Crosslinked.

Remarkably, these release studies were performed almost exclusively at pH 1.2, in either simulated gastric fluid (SGF) or dilute HCl solutions (ca. 0.1 N). It is noteworthy that, although this is the pH of stomach under fasting conditions, it will not be the pH encountered in the surrounding of *H. pylori-*infested stomach tissue, which leads to higher pH figures than those found in healthy, empty stomachs [88]. This is not a trivial issue because AMOX is a drug with two ionizable groups [AMOX p*k*_a_ (strongest basic): 7.22; AMOX p*k*_a_ (strongest acidic): 3.23, values obtained from www.drugbank.com, (accessed on 25 May 2022)] and, hence, its release profile will vary with gastric pH. Moreover, the co-administration of proton pump inhibitors or P-CAB in anti-*H. pylori* therapies raises the pH of gastric fluids to values ≥4.0. Consequently, it may happen that apparently successful formulations will not work in in vivo studies. Similarly, the behaviors of pH-responsive materials used in the development of such GRDDSs (such as NaAlg and G, for example) are expected to be different from those observed at pH 1.2.

The other aspect to consider is the variety of drug cargo in each system, which will determine the final area under the curve (AUC) for AMOX and, therefore, the amount of drug available in the stomach to exert its antibacterial action. Despite the relevance of this information, it is not usually included explicitly in the papers. On another note, there are a few drawbacks generally associated with floating GRDDSs that must be overcome for the optimal final performance of these pharmaceutical formulations. Since the drug and the low-density system itself remain so intricately joined when the formulation is generated, one cannot be changed without the other, that is, release kinetics cannot be modified without irremediably varying the buoyancy of the system. Moreover, these kinds of formulation may tend to stick together when administered, potentially causing bowel obstruction, and they need high levels of fluid to work optimally [76].

To sum up, the design of systems capable of loading large quantities of AMOX, performing at different pHs (1.2 and higher), and being stable and capable of disintegrating when the action is exerted is of fundamental importance. Insufficient research has been conducted in the pursuit of new floating materials capable of imparting mechanical stability and at the same time being swellable, porous, and degradable. There is no example in which the floatability of AMOX-loaded GRDDS is determined by its own microstructure. Additional properties, such as mucoadhesion and the capability to encapsulate large drug loads, will be of great significance. The development of such dosage forms is yet to be demonstrated.

### 7.3. Mucoadhesive Formulations

The other highly convenient type of GRDDS is mucoadhesive formulations. This is an excellent option when the pharmacological action required is local, since the mucoadhesive GRDDS can come into close contact with the infected area, remain adhered to the gastric mucosa for a prolonged period, and, consequently, achieve a sustained release of the drug where the pathogen is located.

Mucus is a viscoelastic, gel-like, stringy slime which is mainly constituted of water (≤95% weight), inorganic salts (~1% weight), carbohydrates and lipids (<1%), and glycoproteins (<5% weight, also called mucins) [92]. The primary function of gastric mucus is to protect the gastric epithelium from acid and peptidases. In addition, it serves as a lubricant for the passage of solids and as a barrier to antigens, bacteria, and viruses. The epithelial adhesive properties of mucin are well known and have been applied to the development of GRDDSs through the use of bio/mucoadhesive polymers [93].

Mucoadhesion can be defined as a characteristic feature of some natural or synthetic polymers that are able to attach to a mucosal surface. The process that evolves towards the mucoadhesive phenomenon can be described in three consecutive steps. (a) Wetting and swelling of the polymer is the initial step, which progresses to intimate contact with the mucosa. Hydrophilic polymers tend to absorb large amounts of water and become sticky, thus acquiring bioadhesive properties. (b) Once the hydrated polymer is in close contact with the mucosa, interpenetration and entanglement between the polymer and the mucin chains take place. These are physical-mechanical bonds that are related to the flexibility of the polymer chains and frequently occur in polysaccharide-based gums. (c) Lastly, this intricate intertwining of chains promotes the formation of secondary chemical bonds, i.e., electrostatic and hydrogen bonding, the latter being one of the most relevant in this type of interaction [92].

Considering this mechanism, mucoadhesive properties can be enhanced, firstly, by the presence of hydrophilic functional groups that could promote the swelling process. Adhesion has been shown to be enhanced by rapid hydration, and there is linear relationship between swelling index and mucoadhesion, as demonstrated in some GRDDS formulations [94]. Secondly, the flexibility of the polymer chains promotes the final mucoadhesive performance and, thirdly, the existence of ionic groups or/and proton donor or acceptor groups (such as hydroxyl, carboxyl, sulfate, and amino groups) is beneficial as they can participate in the formation of electrostatic or/and hydrogen bonds. Interestingly, polymers functionalized with sulfhydryl groups can render reversible disulfide linkages with mucin [95].

When eradication of *H. pylori* infection is aimed at, the preparation of mucoadhesive, gastroretentive dosage forms is especially advantageous, as they can adhere to the stomach wall, survive the gastrointestinal motility for a longer period [96], and thus enhance the local action of the drug in the infected area [97].

Polymers with high mucoadhesive strength are required for successful design of the mucoadhesive dosage form. Mucoadhesive polymers such as chitosan (CTS), pectin, Carbopol^®^, polyacrylic acid (PAA), and sodium carboxymethylcellulose (NaCMC) were reported as materials of interest in the preparation of mucoadhesive GRDDSs, as well as gums such as guar gum, gellan gum, and xanthan gum, and thiolated polymers, among others (Table 3).

These polymers are characterized by their molecular flexibility and the presence of hydrophilic functional groups in their structure; they are non-toxic, non-absorbable, and inexpensive. Their interaction with mucin–epithelial surfaces is by means of non-covalent bonds, and they also readily adhere to moist surfaces. Another of their most significant features is that they can easily encapsulate a variety of drugs and not hinder the release of APIs, as will be demonstrated next.

However, the main inconvenience to overcome for optimal final performance of mucoadhesive pharmaceutical formulations is related to their proper adhesion to the stomach mucosa, which is directly correlated with their final performance. In this way, the constant turnover of this protective mucus layer could reduce the bioadhesion of the polymers. In this case, the use of microscale mucoadhesive particles, could be of help.

### 7.4. Micro and Nanostructured Mucoadhesive GRDDSs

Micro- or nano-scaled mucoadhesive particles, such as liposome, polymeric, and metallic nanoparticles that may diffuse through the stomach mucosa and reach *H. pylori* have recently emerged as promising delivery mechanisms to improve bacterial eradication efficacy [100,106,109]. In general terms, mucoadhesive microspheres ameliorated the gastric stability of AMOX due to entrapment within the microsphere [103].

Mucoadhesive AMOX-loaded micro- and nanospheres are prepared with the concourse of one or various bioadhesive polymers (Table 4) by two main methods: ionic gelation and emulsion solvent evaporation. The first one (ionic gelation method) is based on the electrostatic interaction between a cationic polymer (CTS [98,104] or its derivatives [110]) with an anionic polysaccharide, typically NaAlg [98,104], PAA [100], sodium carboxymethylcellulose (NaCMC [104]), pectin [106], or poly(malic acid) [110]. Since anionic polymers are polycarboxylic materials, Ca^2+^ ions from CaCl_2_ or CaSO_4_ can be added to improve the overall stability of the formulations [98,104,105]. Similarly, sulfate anions from Na_2_SO_4_ or CaSO_4_ are employed to strengthen CTS-based GRDDSs [98,105]. CTS is frequently employed as a coating agent [98,104]. On the other hand, for the emulsion solvent evaporation method, a lipid phase is necessary (light liquid paraffin (LLP) [109] or liquid paraffin (LP) [102,103]), as well as a surfactant (for example, Span^®^ 80 [102,109] or Poloxamer^®^ 188 [73]). Microsphere formation has also been made by spray-drying method using CTS as the only mucoadhesive polymer, which was chemically crosslinked with glutaraldehyde to impart the required stability to the formulation [99]. The anionic polymer required for these formulations can be prepared de novo, as was the case with the *co*-polymerization of *N*-isopropylamide (NIPAM), acrylic acid (AA) and 2-hydroxyethyl methacrylate (HEMA) for the design of a nanoparticulate formulation in which triethylene glycol dimethacrylate (TEGDMA) was chosen as the chemical crosslinker [73].

Another approach is the preparation of nanostructured formulations such as polymeric nanoparticles [112] and liposomes [106] containing AMOX. The design of mucoadhesive liposomes has been conducted with success by the preparation of pectin-coated liposomes. This formulation is characterized by its ability to diminish the first step in the development of *H. pylori* pathogenicity, i.e., its adhesion to the gastric epithelium [106]. To note, recent studies are focused on nano- and micro-composites for the release of AMOX in which insoluble materials such as magnesium aluminum silicate (MAS, [104]), carbon quantum dots (CDs, [110]) and superparamagnetic iron oxide nanoparticles (SPIO, [100]) are incorporated with the aim of imparting additional properties to the final formulations. Although micro- or nano-scale mucoadhesive particles may diffuse through the stomach mucosa and reach *H. pylori*, they are characterized by low drug-loading capacity [113].

Mucoadhesive performance is commonly measured on model mucosae (goat intestinal mucosa [109], pig’s ileum [98], or rat stomach mucosa [99,102,103]) or on freshly prepared simulated gastric mucosa [105]. In another study [88], Wistar rats were employed to study in vivo gastric mucoadhesion. The percentage of AMOX-loaded microparticles that remain adhered to the tissue is evaluated, either for a pre-established time or after washing them out with simulated gastric or intestinal fluids (SGF or SIF, respectively). Other methods involve the use of commercial mucins, and bioadhesive properties are determined by microviscometry [106] or based on changes in parameters such as surface charge potentials [100] and aggregation rate [110] (Table 4).

A linear relationship was observed between the swelling index of microspheres and their mucoadhesion [94], apart from the impact that the former exerted on drug release, as will be discussed below. Therefore, determination of swelling properties is crucial: massive increases in weight have been found for some formulations such as *co*-p(NIPAM-AA-HEMA) nanoparticles (NPs, swelling index: 900–1300%) [73], composite blend microbeads of NaAlg, NaCMC and MAS particles (swelling index: 280–730%) [104], minimatrices of xanthan gum (XG), HPMC and PEO (swelling index: 150–500%) [87], and microspheres made of NaAlg, guar gum (GG), and Carbopol^®^ (swelling index: 55–260%) [94], to name a few examples. The degree of crosslinking of the prepared microspheres is directly connected with their mucoadhesive properties, so the higher the degree of crosslinking of the materials, the lower their swelling parameters, and hence, a consequent reduction in mucoadhesive properties will be found [99].

Swelling properties were also investigated to assess their influence on solvent uptake and, thus, release patterns, and a direct relationship with drug diffusion was observed [94]. For instance, it has been reported that the inclusion of guar gum in the composition of AMOX-loaded formulations facilitated the transfer of solvent to the microspheres. The observed sustained drug release was attribute to guar gum hydration and swelling. A similar behavioral pattern was found in chitosan microspheres, and a linear correlation between swelling index and the in vitro AMOX release was established [99]. The degree of swelling was likewise negatively linked to the degree of crosslinking [99,109]. Furthermore, in MAS-based composite blend microbeads, the higher the swelling, the higher the experimental release rate [104]. The release of the drug was also affected by the polymer concentration (negative outcome) [103] and the presence of a lipophilic excipient (for example, sunflower oil), which formed a strong hydrophobic diffusional barrier and retarded the drug release from the beads significantly [72].

In contrast to release studies conducted with floating GRDDSs, the release behavior of AMOX was investigated for some mucoadhesive systems not only at pH 1.2, but also at pH 5.0 [105] or pH ca. 7.0 [73,98,110] (simulating gastric acid, gastric mucosa, or optimal *H. pylori* survival conditions, respectively). Sequential pH changes throughout the experiment were also addressed for magnetic and CDs-based nanocomposites [100,110]. In general terms, in vitro AMOX release rates were higher at pH ≈ 1 than those found at pH ≈ 7 [73,98].

However, as with floating GRDDSs, there are some inconveniences to overcome for optimal final performance of mucoadhesive pharmaceutical formulations. The main aspect to deal with is related to their proper adhesion to the stomach mucosa, which is directly correlated with their final performance. In this way, the constant turnover of this protective mucus layer could reduce the bioadhesion of the polymers.

Consequently, although the development of simple-working GRDDSs has partially helped to overcome the drawbacks associated with conventional dosage forms, further work is needed on its shortcomings. The development of dual-working polymeric materials (such as floating and mucoadhesive systems) in the treatment of *H. pylori* infections is an interesting approach to substantially lengthen the gastric residence time of AMOX (Table 5 includes some examples of such systems). Giving buoyant properties to mucoadhesive multiparticulate dosage forms may guarantee consistent release of the drug in the target site. The portion of beads that remains floating will be available to ensure the replenishment of the units detached due to the turnover of the mucus layer.

## 8. Conclusions

In summary, an in-depth study on *H. pylori* infections, as well as the current status and the difficulties encountered in its eradication, have been the central topics of the present work. Many attempts have been made to prepare GRDDSs with suitable properties for application as anti-*H. pylori* treatments, which is especially relevant due to the relationship established between this pathogen and gastric cancer. Amoxicillin has proven to be the most effective antibiotic and the one showing the least amount of resistant *H. pylori* strains within the therapeutic arsenal against this bacterium. Numerous buoyant and mucoadhesive GRDDSs, as well as dual-working systems, have been designed and formulated. This review analyzes the most relevant works related to them, highlighting the main strategies followed and the advantages and drawbacks associated with them. The procedures described herein could shed some light on the development of novel and better-tuned materials against the colonization of this microorganism. We anticipate that the present work could inspire the development of cutting-edge GRDDSs with clear and improved benefits in *H. pylori* treatments that may extend to improving therapeutic approaches for other endemic diseases.

## Figures and Tables

**Table 3:** Classification of bioadhesive polymers used in the treatment of infections by *H. pylori*.

Type of Mucoadhesive Polymer	Polymer	Ref.
Cationic polymers	CTS	[72,98,99]
(Semi)synthetic anionic polymers	PAA	[73,100]
	Carbopol^®^	[90,101,102,103]
	Polycarbophil^®^	[101]
	NaCMC	[104]
Anionic polysaccharides and gums	NaAlg	[98,105]
	Pectin	[106,107]
	Gellan gum	[90]
	Xanthan gum	[105,108]
Other polysaccharides and gums	Guar gum	[94,108]
	Gum ghatti	[108]
	Sterculia foetida	[109]
	Pullulan	[109]
Thiolated polymers	Thiolated CTS	[110]
	Thiolated PAA	[111]

Carbopol^®^: PAA; CTS: Chitosan; NaAlg: Sodium alginate; NaCMC: Sodium carboxymethyl cellulose; PAA: Polyacrylic acid; Polycarbophil^®^: PPA.

**Table 4:** Selected AMOX-loaded bioadhesive formulations used in the treatment of infections by *H. pylori*.

Drug	Formulations	Matrix-Forming Polymers	Other Components	Mucoadhesive Performance	DL EE	Sustained Release: Time (h), and Cumulative Drug Release (%)	Method	Ref.
AMOX	Semi-IPN microspheres	*Sterculia foetida* Pullulan	- LLP (lipid phase), - Span 80 (surfactant) - Glutaraladehyde (Xrlinker)	Mucoadh. (goat intestinal mucosa): 81.7% at 12 h	- … - EE: 60–88%	pH 1.2: 60–89% in 12 h	Water-in-oil emulsification-crosslinking method	[109]
AMOX	Mucoadhesive microparticles	NaAlg CTS as coating agent	CaCl_2_ (XrL NaAlg) Na_2_SO_4_ (XrL CTS)	Mucoadh. (pig’s ileum): 76%, 100 mL SIF	- -- - EE: 96–97%	- SGF: 28–45% in 24 h - SIF: 21–39% in 24 h	Ionic gelation method	[98]
AMOX	Mucoadhesive microspheres	Carbopol^®^ 934P EC	LP (lipid phase) Span 80 (surfactant)	Mucoadh. (rat stomach mucosa): 48–68% for 10 h	- … - EE: 66%	- pH 1.2 and - pH 7.8: *ca*. 90% in 10 h	Emulsion solvent evaporation method	[102]
AMOX	Mucoadhesive microspheres	Carbopol^®^ 974P, HPMC K4M, Eudragit RS 100	LP (lipid phase)	Mucoadh. (rat stomach mucosa): 56–89% at 6 h	- DL: 5% - EE: 57–88%	pH 1.2: Initial burst effect 75–100% in 7 h	Microspheres were prepared by solvent evaporation technique	[103]
AMOX	Mucoadhesive microspheres	CTS	Glutaraladehyde (Xrlinker)	Mucoadh. (rat stomach mucosa): 38–62% after 5 h	- DL: 25–50% - EE: 77–92%	pH: 1.2 It was measured the time required for 80% release: 3–10 h	Spray-drying method followed by chemical XrL (glutaraldehyde)	[99]
AMOX	Nanocomposites of CDs and CTS-based NP	Thiolated- ureido-CTS (mucoadh) Poly(malic acid)	CDs	Mucoadh. Measured as aggregation rate with mucin (fluorescence intensity after 3 h): 19–46%	- DL: 24–28% - …	Changing pH along the experiment (final time 12 h) - pH 1.2 (2 h): 35% - pH 6.0 (2 h): 60% - pH 7.0, 8 h: 80–95%	- CTS-based NP: ionic gelation method - CD-NP Composite: chemical reaction (Amide formation) between CDs and NP	[110]
AMOX	Composite blend microbeads	NaAlg NaCMC	- MAS particles - Chitosan (enteric coated) - CaCl_2_	…	- … - EE: 52–92%	- pH 1.2 20–40% in 8 h (depending on matrix swelling).	Ionic gelation method	[104]
AMOX	Magnetic NP	CTS, PAA	SPIO	Mucoadhesion determined by changes in the surface charge potential of mucin particles upon absorption of mucoadhesive polymer	- DL: 0.1% (*w*/*v*) - EE: 77.8%	Changing pH along the experiment (final time 24 h) - pH 2.5 (2 h): *ca*. 20% - pH 6.5 (2 h): 30–40% in 4 h - pH 7.4 (20 h): 70%	Ionic gelation method	[100]
AMOX	Uncoated or pectin-coated liposomes (UCL, CL)	Pectin as coating agent	Lecithin, cholesterol, DDAB	Mucoadh. Measured by microviscometry (MX of UCL and CL with pig gastric mucin type III).	- … - EE: - UCL: 66% - CL: 83%	pH not stated - UCL: 85% (= 6.1 μM) in 1 h - CL: 75% (=62.3 μM) in 1 h	Thin-film hydration method	[106]
AMOX	Xrlinked hydrogel NPs	Monomers used: NIPAM (thermoresponsive) + AA (bioadhesive) + HEMA	BPO (radical initiatior) XrLinker: TEGDMA Poloxamer 188 (surfactant for emulsion-evaporation method)	…	- DL: 1–2% - EE: 70.2–91.4%	- pH 1.0: 88.5% in 4 h - pH 7.4: 45% in 4 h	- Synthesis of polymer: Radical polymerization - NP formation: Emulsion-evaporation technique	[73]
AMOX CLA	Liquid hydrogel	MUCOLAST^®^: NaAlg, NaCMC, XG	CaSO_4_ (XrLinker) Glycerol/GMS as drug solvents	Mucoadh. Time needed to detach formulation from simulated gastric mucosa: 4.0–6.7 h	- DL: - AMOX: 89.4–8.94 mg/100 μL - CLA: 44.7–4.47 mg/100 μL	pH: 5.0 AMOX: 50–60% CLA: 35–40%	Ionic gelation method	[105]

AA: Acrylic acid; AMOX: Amoxicillin; BPO: Benzoyl peroxide; Carbopol^®^: PAA; CDs: Carbon quantum dots; CL: Coated liposomes; CLA: Clarithromycin; CTS: Chitosan; DDAB: Didodecyldimethylammonium bromide; DL: Drug loading; EC: Ethyl cellulose; EE: Encapsulation efficiency; GMS: Glyceryl monostearate; HEMA: 2-Hydroxyethyl methacrylate; HPMC: Hydroxypropylmethyl cellulose; IPN: Interpenetrated polymer network; LLP: Light liquid paraffin; LP: Liquid paraffin; MAS: Magnesium aluminum silicate; MX: Mixture; NaAlg: Sodium alginate; NaCMC: Sodium carboxymethyl cellulose; NIPAM: *N*-Isopropylamide; NP: Nanoparticles; PAA: Polyacrylic acid; SGF: Simulated gastric fluid; SIF: Simulated intestinal fluid; SPIO: Superparamagnetic iron oxide nanoparticles; TEGDMA: Triethyleneglycol dimethacrylate; UCL: Uncoated liposomes; XG: Xanthan gum; XrL: Crosslinked; XrLinker: Crosslinker.

**Table 5:** Selected AMOX-loaded floating and mucoadhesive formulations used in the treatment of infections by *H. pylori*.

Drug	Formulation	Matrix-Forming Polymers	Other Components	Floating Time (FT) Floating Lag Time (FLT) Mucoadhesive Performance	DL EE	Sustained Release: Time (h), and Cumulative Drug Release (%)	Method	Ref.
AMOX	Coated oil-entrapped beads	NaAlg HPMC	- CTS (coating and mucoadh.) - Sunflower oil -CaCl_2_	FT > 24 h - FLT:43–50 s - Mucoad. (Sheep stomach mucosa): 75–85%	… 55.2–90.9%	pH 1.0: 59–78% in 7 h	Ionotropic gelation method	[72]
AMOX	Microspheres	CPG NaAlg	- CTS (coating and mucoadh.) - CaCO_3_ - CaCl_2_	- Float. Capac.: 72–87% - FLT: 4–10 min - Mucoadh (in vivo, Wistar rats): 85%, 7 h	-- 65–89%	pH 1.2: 79–92% in 8 h	Ionotropic gelation method	[88]
Mz	Floating and mucoadh. microspheres	NaAlg, GG Carbopol^®^	CaCl_2_, NaHCO_3_, Eudragit^®^ L100	- FT > 8 h … - Mucoadh. (rat stomach mucosa): 61%	… 40–76%	pH 1.2: 29–73% in 8 h	Ionotropic gelation method	[94]
AMOX	Oil-entrapped buoyant beads	G HPMC or Carbopol^®^ 934	- Light mineral oil - CaCO_3_, CaCl_2_ - EC (coating)	Float. Capac.: 60–85% … …	48–82% 73–96%	pH 1.2: 76% in 8 h pH 3.4: 62% in 8 h	Ionotropic gelation method	[90]
AMOX	Minimatrices	XG HPMC K100M CR/PEO	- NaHCO_3_ - Citric acid - Carbopol^®^ 974P (lubricant and mucoadhesive) PVP K30	- FT > 12 h - FLT: 7–32 min - Bioadhesive strength (goat stomach tissue): 5.6–18 dyn/cm^2^		pH 1.2: 31.9–53.3% in 1 h 95% in 2.6–9.4 h	Non aqueous granulation method	[87]

AMOX: Amoxicillin; Carbopol^®^: PAA; CPG*: Caesalpinia pulcherrima* galactomannan; CTS: Chitosan; DL: Drug loading; EC: Ethyl cellulose; EE: Encapsulation efficiency; FLT: Floating lag time; FT: Floating time; G: Gellan gum; GG: Guar gum; HPMC: Hydroxypropylmethyl cellulose; Mz: Metronidazole; NaAlg: Sodium Alginate; NaCMC: Sodium carboxymethyl cellulose; PVP: Polyvinyl pyrrolidone; XG: Xanthan gum.

## Abbreviations

AA: Acrylic acid; AMOX: Amoxicillin; API: Active pharmaceutical ingredient; AUC: Area under the curve; BabA: Blood group antigen-binding adhesin; BPO: Benzoyl peroxide; cagPAI: Cag-pathogenicity island; CAP: Cellulose acetate phtalate; CAP: Cellulose acetate phtalate; Carbopol^®^: PAA; CDs: Carbon quantum dots; CGT: Cholesterol glycosyl-transferase; CL: Coated liposomes; CLA: Clarithromycin; COM: Compritol^®^; CPG: *Caesalpinia pulcherrima* galactomannan; Cr: Compritol ATO 888; CTS: Chitosan; CYP2C19: cytochrome P450 isoenzyme 2C19; DDAB: Didodecyldimethylammonium bromide; DL: Drug loading; DDS: Drug delivery system; EC: Ethyl cellulose; EE: Encapsulation efficiency; FAM: Famotidine; FLT: Floating lag time; FRS: Floating raft system; G: Gellan gum; GG: Guar gum; GIT: Gastrointestinal tract; GMS: Glyceryl monostearate; GRDDS: Gastroretentive drug delivery system; GRT: Gastric residence time; HEMA: 2-Hydroxyethyl methacrylate; HopQ: *Helicobacter pylori* outer membrane protein; HPMC: Hydroxypropylmethyl cellulose; HtrA: High-temperature requirement protein; KG: Karaya gum; Kolliphor^®^HS 15: Mixture of free PEG 660 and PEG 660- 12-hydroxystearate; Labrafac™ WL1349: Caprylic/capric acid triglycerides medium-chain triglycerides; Labrasol^®^ ALF: PEG-8 caprylic/capric glycerides; LLP: Light liquid paraffin; LNC: Lipid nanocapsules; LP: Liquid paraffin; LPN: Lipid polymer nanoparticles; MALToma: Gastric mucosa-associated lymphoid tissue lymphoma; MAS: Magnesium aluminum silicate; MC: Microcapsule; MCC: Microcrystalline cellulose; MIC: Minimum inhibitory concentration; MTs: Minitablets; MX: Mixture; Mz: Metronidazole; NaAlg: Sodium alginate; NaCMC: Sodium carboxymethyl cellulose; NIPAM: *N*-isopropylamide; OipA: Outer inflammatory protein; PAA: Polyacrylic acid; P-CAB: Potassium-competitive acid blocker; Polycarbophil^®^: PAA; PRE: Precirol^®^; PVA: Polyvinyl alcohol; PVP: Polyvinyl pyrrolidone; SabA: Sialic acid-binding adhesin; SGF: Simulated gastric fluid; SIF: Simulated intestinal fluid; SPIO: Superparamagnetic iron oxide nanoparticles; TAKECAB^®^: Formulation for vonoprazan sold in Japan; Talicia^®^: Delayed-release capsules with rifabutin, omeprazole, magnesium, and amoxicillin; TEGDMA: Triethyleneglycol dimethacrylate; T4SS: Type IV secretion system; UCL: Uncoated liposomes; VacA: Vacuolating cytotoxin; VONO: Vonoprazan fumarate; XG: Xanthan gum; XrL: Crosslinked.

## References

[1] Institute for Health Metrics and Evaluation (IHME) (2019). GBD Compare.

[2] Goodwin C.S., Armstrong J.A., Chilvers T., Peters M., Collins M.D., Sly L., McConnell W., Harper W.E.S. (1989). Transfer of Campylobacter pylori and Campylobacter mustelae to Helicobacter gen. nov. as Helicobacter pylori comb. nov. and Helicobacter mustelae comb. nov., respectively. Int. J. Syst. Bacteriol..

[3] Cardos I.A., Zaha D.C., Sindhu R.K., Cavalu S. (2021). Revisiting therapeutic strategies for h. Pylori treatment in the context of antibiotic resistance: Focus on alternative and complementary therapies. Molecules.

[4] Stark R.M., Gerwig G.J., Pitman R.S., Potts L.F., Williams N.A., Greenman J., Weinzweig I.P., Hirst T.R., Millar M.R. (1999). Biofilm formation by Helicobacter pylori. Lett. Appl. Microbiol..

[5] American Cancer Society (2021). Cancer Facts & Figures 2021.

[6] Alam J., Dilnawaz F., Sahoo S., Singh D., Mukhopadhyay A., Hussain T., Pati S. (2021). Curcumin Encapsulated into Biocompatible Co-Polymer PLGA Nanoparticle Enhanced Anti-Gastric Cancer and Anti-Helicobacter Pylori Effect. Asian Pac. J. Cancer Prev..

[7] Pasechnikov V., Chukov S., Fedorov E., Kikuste I., Leja M. (2014). Gastric cancer: Prevention, screening and early diagnosis. World J. Gastroenterol..

[8] Fernández-Navarro P., Roquette R., Nuñez O., de Sousa-Uva M., García-Pérez J., López-Abente G., Nunes B., González-Sánchez M., Dinis J., Carmona R. (2021). Atlas of Cancer Mortality in Portugal and Spain (2003–2012).

[9] Machlowska J., Baj J., Sitarz M., Maciejewski R., Sitarz R. (2020). Gastric Cancer: Epidemiology, Risk Factors, Classification, Genomic Characteristics and Treatment Strategies. Int. J. Mol. Sci..

[10] Cheung K.S., Chan E.W., Wong A.Y.S., Chen L., Seto W.K., Wong I.C.K., Leung W.K. (2018). Aspirin and Risk of Gastric Cancer After Helicobacter pylori Eradication: A Territory-Wide Study. JNCI J. Natl. Cancer Inst..

[11] Hansson L.-E., Nyrén O., Hsing A.W., Bergström R., Josefsson S., Chow W.-H., Fraumeni J.F., Adami H.-O. (1996). The risk of stomach cancer in patients with gastric or duodenal ulcer disease. N. Engl. J. Med..

[12] Lee Y.-C., Dore M.P., Graham D.Y. (2022). Diagnosis and Treatment of Helicobacter pylori Infection. Chin. J. Gastroenterol..

[13] Programas de Cribado de Cáncer Ministerio de Sanidad (Gobierno de España). https://www.sanidad.gob.es/profesionales/saludPublica/prevPromocion/Cribado/cribadoCancer.htm.

[14] Goral V. (2016). Etiopathogenesis of Gastric Cancer. Scand. J. Surg..

[15] Wroblewski L.E., Peek R.M., Wilson K.T. (2010). Helicobacter pylori and gastric cancer: Factors that modulate disease risk. Clin. Microbiol. Rev..

[16] Yang J.C., Lu C.W., Lin C.J. (2014). Treatment of Helicobacter pylori infection: Current status and future concepts. World J. Gastroenterol..

[17] Gu H. (2017). Role of Flagella in the Pathogenesis of Helicobacter pylori. Curr. Microbiol..

[18] Sankararaman S., Moosavi L. (2022). Urea Breath Test. StatPearls.

[19] Sgouras D., Tegtmeyer N., Wessler S. (2019). Activity and Functional Importance of Helicobacter pylori Virulence Factors. Adv. Exp. Med. Biol..

[20] Goderska K., Agudo Pena S., Alarcon T. (2018). Helicobacter pylori treatment: Antibiotics or probiotics. Appl. Microbiol. Biotechnol..

[21] Hooi J.K.Y., Lai W.Y., Ng W.K., Suen M.M.Y., Underwood F.E., Tanyingoh D., Malfertheiner P., Graham D.Y., Wong V.W.S., Wu J.C.Y. (2017). Global Prevalence of Helicobacter pylori Infection: Systematic Review and Meta-Analysis. Gastroenterology.

[22] Shichijo S., Hirata Y. (2018). Characteristics and predictors of gastric cancer after Helicobacter pylori eradication. World J. Gastroenterol..

[23] Ansari S., Yamaoka Y. (2019). Helicobacter pylori virulence factors exploiting gastric colonization and its pathogenicity. Toxins.

[24] Kao C.Y., Sheu B.S., Wu J.J. (2016). Helicobacter pylori infection: An overview of bacterial virulence factors and pathogenesis. Biomed. J..

[25] Terebiznik M.R., Raju D., Vázquez C.L., Torbricki K., Kulkarni R., Blanke S.R., Yoshimori T., Colombo M.I., Jones N.L. (2009). Effect of Helicobacter pylori’s vacuolating cytotoxin on the autophagy pathway in gastric epithelial cells. Autophagy.

[26] Gorbach S.L. (1990). Bismuth therapy in gastrointestinal diseases. Gastroenterology.

[27] Guevara B., Cogdill A.G. (2020). Helicobacter pylori: A Review of Current Diagnostic and Management Strategies. Dig. Dis. Sci..

[28] Keller A. (2020). Fluoroquinolones. Consumer Notice. https://www.consumernotice.org/drugs-and-devices/fluoroquinolones/.

[29] Kiyotoki S., Nishikawa J., Sakaida I. (2020). Efficacy of vonoprazan for helicobacter pylori eradication. Intern. Med..

[30] Scott D.R., Sachs G., Marcus E.A. (2016). The role of acid inhibition in Helicobacter pylori eradication [version 1; referees: 3 approved]. F1000Research.

[31] Furuta T., Graham D.Y. (2012). Pharmacologic Aspects of Eradication Therapy for Helicobacter pylori Infection. Gastroenterol. Clin. N. Am..

[32] Reed M.D. (2000). Optimal antibiotic dosing. The pharmacokinetic-pharmacodynamic interface. Postgrad. Med..

[33] Gisbert J.P. (2020). Empirical or susceptibility-guided treatment for Helicobacter pylori infection? A comprehensive review. Ther. Adv. Gastroenterol..

[34] Chey W.D., Leontiadis G.I., Howden C.W., Moss S.F. (2017). ACG Clinical Guideline: Treatment of Helicobacter pylori Infection. Am. J. Gastroenterol..

[35] Guo B., Cao N.W., Zhou H.Y., Chu X.J., Li B.Z. (2021). Efficacy and safety of bismuth-containing quadruple treatment and concomitant treatment for first-line Helicobacter pylori eradication: A systematic review and meta-analysis. Microb. Pathog..

[36] Li B.Z., Threapleton D.E., Wang J.Y., Xu J.M., Yuan J.Q., Zhang C., Li P., Ye Q.L., Guo B., Mao C. (2015). Comparative effectiveness and tolerance of treatments for Helicobacter pylori: Systematic review and network meta-analysis. BMJ.

[37] Malfertheiner P., Megraud F., O’Morain C., Gisbert J.P., Kuipers E.J., Axon A., Bazzoli F., Gasbarrini A., Atherton J., Graham D.Y. (2017). Management of Helicobacter pylori Infection-the Maastricht V/Florence Consensus Report. Gut.

[38] Batista A.D., Rodrigues D.A., Figueiras A., Zapata-Cachafeiro M., Roque F., Herdeiro M.T. (2020). Antibiotic dispensation without a prescription worldwide: A systematic review. Antibiotics.

[39] Graham D.Y., Lu H., Yamaoka Y. (2007). A report card to grade Helicobacter pylori therapy. Helicobacter.

[40] Jaka H., Rhee J.A., Östlundh L., Smart L., Peck R., Mueller A., Kasang C., Mshana S.E. (2018). The magnitude of antibiotic resistance to Helicobacter pylori in Africa and identified mutations which confer resistance to antibiotics: Systematic review and meta-analysis. BMC Infect. Dis..

[41] Schubert J.P., Gehlert J., Rayner C.K., Roberts-Thomson I.C., Costello S., Mangoni A.A., Bryant R.V. (2021). Antibiotic resistance of Helicobacter pylori in Australia and New Zealand: A systematic review and meta-analysis. J. Gastroenterol. Hepatol..

[42] Savoldi A., Carrara E., Graham D.Y., Conti M., Tacconelli E. (2018). Prevalence of Antibiotic Resistance in Helicobacter pylori: A Systematic Review and Meta-analysis in World Health Organization Regions. Gastroenterology.

[43] Bujanda L., Nyssen O.P., Vaira D., Saracino I.M., Fiorini G., Lerang F., Georgopoulos S., Tepes B., Heluwaert F., Gasbarrini A. (2021). Antibiotic resistance prevalence and trends in patients infected with helicobacter pylori in the period 2013–2020: Results of the european registry on h. pylori management (hp-eureg). Antibiotics.

[44] Kasahun G.G., Demoz G.T., Desta D.M. (2020). Primary Resistance Pattern of Helicobacter pylori to Antibiotics in Adult Population: A Systematic Review. Infect. Drug Resist..

[45] Van Khien V., Thang D.M., Hai T.M., Duat N.Q., Khanh P.H., Ha D.T., Binh T.T., Dung H.D.Q., Trang T.T.H., Yamaoka Y. (2019). Management of antibiotic-resistant helicobacter pylori infection: Perspectives from Vietnam. Gut Liver.

[46] Andreev D.N., Maev I.V., Kucheryavyy Y.A. (2020). Helicobacter pylori resistance in the Russian Federation: A meta-analysis of studies over the past 10 years. Ter. Arkhiv.

[47] Khademi F., Sahebkar A. (2020). An Updated Systematic Review and Meta-Analysis on the Helicobacter pylori Antibiotic Resistance in Iran (2010–2020). Microb. Drug Resist..

[48] Li S.Y., Li J., Dong X.H., Teng G.G., Zhang W., Cheng H., Gao W., Dai Y., Zhang X.H., Wang W.H. (2021). The effect of previous eradication failure on antibiotic resistance of Helicobacter pylori: A retrospective study over 8 years in Beijing. Helicobacter.

[49] De Francesco V., Giorgio F., Hassan C., Manes G., Vannella L., Panella C., Ierardi E., Zullo A. (2010). Worldwide H. pylori antibiotic resistance: A systematic review. J. Gastrointest. Liver Dis..

[50] Hafeez M., Qureshi Z.A., Khattak A.L., Saeed F., Asghar A., Azam K., Khan M.A. (2021). Helicobacter Pylori Eradication Therapy: Still a Challenge. Cureus.

[51] Wolle K., Malfertheiner P. (2007). Treatment of Helicobacter pylori. Best Pract. Res. Clin. Gastroenterol..

[52] Phiphatpatthamaamphan K., Vilaichone R.K., Siramolpiwat S., Tangaroonsanti A., Chonprasertsuk S., Bhanthumkomol P., Pornthisarn B., Mahachai V. (2016). Effect of IL-1 polymorphisms, CYP2C19 genotype and antibiotic resistance on Helicobacter pylori eradication comparing between 10-day sequential therapy and 14-day standard triple therapy with four-times-daily-dosing of amoxicillin in Thailand. Asian Pac. J. Cancer Prev..

[53] Hong J., Shu X., Liu D., Zhu Y., Xie C., Xie Y., Zhang K., Wang A., Xiong H., Zeng H. (2016). Antibiotic resistance and CYP2C19 polymorphisms affect the efficacy of concomitant therapies for Helicobacter pylori infection: An open-label, randomized, single-centre clinical trial. J. Antimicrob. Chemother..

[54] Vítor J.M.B., Vale F.F. (2011). Alternative therapies for Helicobacter pylori: Probiotics and phytomedicine. FEMS Immunol. Med. Microbiol..

[55] Astruc B., Jenkins H., Jenkins R. (2017). Effect of Therapeutic and Supratherapeutic Doses of Vonoprazan on the QT/QTc Interval in a Phase I Randomized Study in Healthy Subjects. Clin. Transl. Sci..

[56] Otake K., Sakurai Y., Nishida H., Fukui H., Tagawa Y., Yamasaki H., Karashima M., Otsuka K., Inatomi N. (2016). Characteristics of the Novel Potassium-Competitive Acid Blocker Vonoprazan Fumarate (TAK-438). Adv. Ther..

[57] Mulford D.J., Leifke E., Hibberd M., Howden C.W. (2022). The Effect of Food on the Pharmacokinetics of the Potassium-Competitive Acid Blocker Vonoprazan. Clin. Pharmacol. Drug Dev..

[58] Jenkins H., Sakurai Y., Nishimura A., Okamoto H., Hibberd M., Jenkins R., Yoneyama T., Ashida K., Ogama Y., Warrington S. (2015). Randomised clinical trial: Safety, tolerability, pharmacokinetics and pharmacodynamics of repeated doses of TAK-438 (vonoprazan), a novel potassium-competitive acid blocker, in healthy male subjects. Aliment. Pharmacol. Ther..

[59] Sugano K. (2018). Vonoprazan fumarate, a novel potassium-competitive acid blocker, in the management of gastroesophageal reflux disease: Safety and clinical evidence to date. Ther. Adv. Vaccines.

[60] Echizen H. (2016). The First-in-Class Potassium-Competitive Acid Blocker, Vonoprazan Fumarate: Pharmacokinetic and Pharmacodynamic Considerations. Clin. Pharmacokinet..

[61] Takeda Pharmaceutical Company Limited TAKECAB® Now Available for the Treatment of Acid-Related Diseases in Japan. https://www.takeda.com/newsroom/newsreleases/2015/takecab-now-available-for-the-treatment-of-acid-related-diseases-in-japan/.

[62] Bunchorntavakul C., Buranathawornsom A. (2021). Randomized clinical trial: 7-day vonoprazan-based versus 14-day omeprazole-based triple therapy for Helicobacter pylori. J. Gastroenterol. Hepatol..

[63] Suzuki S., Gotoda T., Kusano C., Ikehara H., Ichijima R., Ohyauchi M., Ito H., Kawamura M., Ogata Y., Ohtaka M. (2020). Seven-day vonoprazan and low-dose amoxicillin dual therapy as first-line Helicobacter pylori treatment: A multicentre randomised trial in Japan. Gut.

[64] Sue S., Shibata W., Sasaki T., Kaneko H., Irie K., Kondo M., Maeda S. (2019). Randomized trial of vonoprazan-based versus proton-pump inhibitor-based third-line triple therapy with sitafloxacin for Helicobacter pylori. J. Gastroenterol. Hepatol..

[65] Scarpignato C., Leifke E., Smith N., Mulford D.J., Lahu G., Facius A., Howden C.W. (2022). A Population Pharmacokinetic Model of Vonoprazan: Evaluating the Effects of Race, Disease Status, and Other Covariates on Exposure. J. Clin. Pharmacol..

[66] Chey W.D., Mégraud F., Laine L., López L.J., Hunt B.J., Howden C.W. (2022). Vonoprazan Triple and Dual Therapy for Helicobacter pylori Infection in the US and Europe: Randomized Clinical Trial. Gastroenterology.

[67] Jenkins H., Jenkins R., Patat A. (2017). Effect of Multiple Oral Doses of the Potent CYP3A4 Inhibitor Clarithromycin on the Pharmacokinetics of a Single Oral Dose of Vonoprazan: A Phase I, Open-Label, Sequential Design Study. Clin. Drug Investig..

[68] Arrua E.C., Sanchez S.V., Trincado V., Hidalgo A., Quest A.F.G., Morales J.O. (2022). Experimental design and optimization of a novel dual-release drug delivery system with therapeutic potential against infection with Helicobacter pylori. Colloids Surf. B Biointerfaces.

[69] Gottesmann M., Goycoolea F.M., Steinbacher T., Menogni T., Hensel A. (2020). Smart drug delivery against Helicobacter pylori: Pectin-coated, mucoadhesive liposomes with antiadhesive activity and antibiotic cargo. Appl. Microbiol. Biotechnol..

[70] Bhattarai N., Gunn J., Zhang M. (2010). Chitosan-based hydrogels for controlled, localized drug delivery. Adv. Drug Deliv. Rev..

[71] Tripathi G.K., Singh S., Nath G., Dubey R.K. (2011). Evaluation of pH Triggers in situ Porous Controlled Release Micro Balloon Delivery of Amoxicillin for Eradication of Helicobacter pylori. Curr. Drug Deliv..

[72] Dey S.K., De P.K., De A., Ojha S., De R., Mukhopadhyay A.K., Samanta A. (2016). Floating mucoadhesive alginate beads of amoxicillin trihydrate: A facile approach for H. pylori eradication. Int. J. Biol. Macromol..

[73] Moogooee M., Ramezanzadeh H., Jasoori S., Omidi Y., Davaran S. (2011). Synthesis and in vitro studies of cross-linked hydrogel nanoparticles containing amoxicillin. J. Pharm. Sci..

[74] Lopes C.M., Bettencourt C., Rossi A., Buttini F., Barata P. (2016). Overview on gastroretentive drug delivery systems for improving drug bioavailability. Int. J. Pharm..

[75] Tripathi J., Thapa P., Maharjan R., Jeong S.H. (2019). Current State and Future Perspectives on Gastroretentive Drug Delivery Systems. Pharmaceutics.

[76] Iglesias N., Galbis E., Romero-Azogil L., Benito E., Lucas R., García-Martín M.G., De-Paz M.-V. (2020). In-Depth Study into Polymeric Materials in Low-Density Gastroretentive Formulations. Pharmaceutics.

[77] Rajinikanth P.S., Balasubramaniam J., Mishra B. (2007). Development and evaluation of a novel floating in situ gelling system of amoxicillin for eradication of Helicobacter pylori. Int. J. Pharm..

[78] Patel D.M., Patel D.K., Patel C.N. (2011). Formulation and Evaluation of Floating Oral In Situ Gelling System of Amoxicillin. ISRN Pharm..

[79] Rajinikanth P.S., Mishra B. (2009). Stomach-site specific drug delivery system of clarithromycin for eradication of Helicobacter pylori. Chem. Pharm. Bull..

[80] Ranade A.N., Wankhede S.S., Ranpise N.S., Mundada M.S. (2012). Development of bilayer floating tablet of amoxicillin and aloe vera gel powder for treatment of gastric ulcers. AAPS PharmSciTech.

[81] Rossi A., Conti C., Colombo G., Castrati L., Scarpignato C., Barata P., Sandri G., Caramella C., Bettini R., Buttini F. (2016). Floating modular drug delivery systems with buoyancy independent of release mechanisms to sustain amoxicillin and clarithromycin intra-gastric concentrations. Drug Dev. Ind. Pharm..

[82] Charoenying T., Patrojanasophon P., Ngawhirunpat T., Rojanarata T., Akkaramongkolporn P., Opanasopit P. (2020). Fabrication of floating capsule-in-3D-printed devices as gastro-retentive delivery systems of amoxicillin. J. Drug Deliv. Sci. Technol..

[83] Kamsali A., Eranti B., Ch M., Manne R., Barghav G.C., Reddy P.S. (2020). Development and Optimization of Amoxicillin Floating Raft System to effectively treat Helicobacter pylori infection. Ars Pharm..

[84] Awasthi R., Kulkarni G.T., Pawar V.K., Garg G. (2012). Optimization studies on gastroretentive floating system using response surface methodology. AAPS PharmSciTech.

[85] Chavanpatil M.D., Jain P., Chaudhari S., Shear R., Vavia P.R. (2006). Novel sustained release, swellable and bioadhesive gastroretentive drug delivery system for ofloxacin. Int. J. Pharm..

[86] Tadros M.I. (2010). Controlled-release effervescent floating matrix tablets of ciprofloxacin hydrochloride: Development, optimization and in vitro–in vivo evaluation in healthy human volunteers. Eur. J. Pharm. Biopharm..

[87] Badhan A.C., Mashru R.C., Shah P.P., Thakkar A.R., Dobaria N.B. (2009). Development and evaluation of sustained release gastroretentive minimatrices for effective treatment of H. pylori Infection. AAPS PharmSciTech.

[88] Thombre N.A., Gide P.S. (2016). Floating-bioadhesive gastroretentive Caesalpinia pulcherrima-based beads of amoxicillin trihydrate for Helicobacter pylori eradication. Drug Deliv..

[89] Abou Youssef N.A.H., Kassem A.A., El-Massik M.A.E., Boraie N.A. (2015). Development of gastroretentive metronidazole floating raft system for targeting Helicobacter pylori. Int. J. Pharm..

[90] Tripathi G.K., Singh S., Nath G. (2012). Formulation and In-vitro evaluation of pH-sensitive oil entrapped polymeric blend amoxicillin beads for the eradication of Helicobacter pylori. Iran. J. Pharm. Res..

[91] Jafar M., Salahuddin M., Khan M.S.A., Alshehry Y., Alrwaili N.R., Alzahrani Y.A., Imam S.S., Alshehri S. (2021). Preparation and in vitro-in vivo evaluation of luteolin loaded gastroretentive microsponge for the eradication of helicobacter pylori infections. Pharmaceutics.

[92] Serra L., Doménech J., Peppas N.A. (2009). Engineering design and molecular dynamics of mucoadhesive drug delivery systems as targeting agents. Eur. J. Pharm. Biopharm..

[93] Nitave S.A., Patil V.A., Kagalkar A.A. (2014). Review on gastro retentive drug delivery system (GRDDS). Int. J. Pharm. Sci. Rev. Res..

[94] Amin M.L., Ahmed T., Mannan M.A. (2016). Development of floating-mucoadhesive microsphere for site specific release of metronidazole. Adv. Pharm. Bull..

[95] Grosso R., De-Paz M.-V. (2021). Thiolated-Polymer-Based Nanoparticles as an Avant-Garde Approach for Anticancer Therapies—Reviewing Thiomers from Chitosan and Hyaluronic Acid. Pharmaceutics.

[96] Zhao S., Lv Y., Zhang J.B., Wang B., Lv G.J., Ma X.J. (2014). Gastroretentive drug delivery systems for the treatment of Helicobacter pylori. World J. Gastroenterol..

[97] Mandal U.K., Chatterjee B., Senjoti F.G. (2016). Gastro-retentive drug delivery systems and their in vivo success: A recent update. Asian J. Pharm. Sci..

[98] Onuigbo E., Onugwu A., Nwocha M., Odiase A., Attama A. (2016). Preparation and in vitro evaluation of amoxicillin encapsulated in alginate-coated chitosan microparticles. Trop. J. Pharm. Res..

[99] Raval J., Patel J., Patel M. (2010). Formulation and in vitro characterization of spray dried microspheres of amoxicillin. Acta Pharm..

[100] Yang S.-J., Huang C.-H., Yang J.-C., Wang C.-H., Shieh M.-J. (2020). Residence Time-Extended Nanoparticles by Magnetic Field Improve the Eradication Efficiency of Helicobacter pylori. ACS Appl. Mater. Interfaces.

[101] Stephin J., Marina K. (2020). Preparation and Investigation of Gastro-Retentive Mucoadhesive Microspheres of Clarithromycin-Resin Complex. Int. J. Pharm. Investig..

[102] Hardenia A., Gupta A.K. (2016). Development and optimization of gastroretentive mucoadhesive microspheres using 33 factorial design. Int. J. Pharm. Sci. Res..

[103] Venkateswaramurthy N., Sambathkumar R., Perumal P. (2011). Design and evaluation of controlled release mucoadhesive microspheres of amoxicillin for anti Helicobacter pylori therapy. Asian J. Pharm..

[104] Angadi S.C., Manjeshwar L.S., Aminabhavi T.M. (2012). Novel composite blend microbeads of sodium alginate coated with chitosan for controlled release of amoxicillin. Int. J. Biol. Macromol..

[105] Villegas I., Rosillo M.Á., Alarcón-de-la-Lastra C., Vázquez-Román V., Llorente M., Sánchez S., Gil A.G., Alcalde P., González E., Rosell E. (2021). Amoxicillin and Clarithromycin Mucoadhesive Delivery System for Helicobacter pylori Infection in a Mouse Model: Characterization, Pharmacokinetics, and Efficacy. Pharmaceutics.

[106] Menchicchi B., Fuenzalida J.P., Bobbili K.B., Hensel A., Swamy M.J., Goycoolea F.M. (2014). Structure of Chitosan Determines Its Interactions with Mucin. Biomacromolecules.

[107] Shastri D.H., Dodiya H.D., Shelat P., Bhanupriy A.K. (2016). Formulation development and evaluation of a gastroretentive in situ oral gel of Cefuroxime Axetil. J. Young Pharm..

[108] Earle R.R., Bharathi V.V., Lakshmi Usha A., Ksheera Bhavani A.V.S. (2020). Cross-Linked Chitosan Based Stomach Specific Mucoadhesive Microspheres Loaded with Amoxicillin: Preparation and ex vivo Characterization. Int. J. Pharm. Investig..

[109] Hadke J., Khan S. (2021). Preparation of sterculia foetida-pullulan-based semi-interpenetrating polymer network gastroretentive microspheres of amoxicillin trihydrate and optimization by response surface methodology. Turk. J. Pharm. Sci..

[110] Arif M., Sharaf M., Samreen, Dong Q., Wang L., Chi Z., Liu C.-G. (2021). Bacteria-targeting chitosan/carbon dots nanocomposite with membrane disruptive properties improve eradication rate of Helicobacter pylori. J. Biomater. Sci. Polym. Ed..

[111] Srivastava A., Verma A., Saraf S., Jain A., Tiwari A., Panda P.K., Jain S.K. (2021). Mucoadhesive gastroretentive microparticulate system for programmed delivery of famotidine and clarithromycin. J. Microencapsul..

[112] Abdelghany A., El-Desouky M.A., Shemis M. (2021). Synthesis and characterization of amoxicillin-loaded polymeric nanocapsules as a drug delivery system targeting Helicobacter pylori. Arab J. Gastroenterol..

[113] Shen S., Wu Y., Liu Y., Wu D. (2017). High drug-loading nanomedicines: Progress, current status, and prospects. Int. J. Nanomed..

